# Image-guided combination chemotherapy and photodynamic therapy using a mitochondria-targeted molecular probe with aggregation-induced emission characteristics†
†Electronic supplementary information (ESI) available. See DOI: 10.1039/c5sc00826c



**DOI:** 10.1039/c5sc00826c

**Published:** 2015-05-18

**Authors:** Chong-Jing Zhang, Qinglian Hu, Guangxue Feng, Ruoyu Zhang, Youyong Yuan, Xianmao Lu, Bin Liu

**Affiliations:** a Department of Chemical and Biomolecular Engineering , National University of Singapore , 4 Engineering Drive 4 , Singapore , 117585 , Singapore . Email: cheliub@nus.edu.sg; b Institute of Materials Research and Engineering , Agency for Science, Technology and Research (A*STAR) , 3 Research Link , Singapore , 117602 , Singapore

## Abstract

Subcellular targeted cancer therapy and *in situ* monitoring of therapeutic effect are highly desirable for clinical applications. Herein, we report a series of probes by conjugating zero (**TPECM-2Br**), one (**TPECM-1TPP**) and two (**TPECM-2TPP**) triphenylphosphine (TPP) ligands to a fluorogen with aggregation-induced emission (AIE) characteristics. The probes are almost non-emissive as molecularly dissolved species, but they can light up in cell cytoplasm or mitochondria. **TPECM-2TPP** is found to be able to target mitochondria, depolarize mitochondria membrane potential and selectively exert potent chemo-cytotoxicity on cancer cells. Furthermore, it can efficiently generate singlet oxygen with strong photo-toxicity upon light illumination, which further enhances its anti-cancer effect. On the other hand, **TPECM-1TPP** can also target mitochondria and generate singlet oxygen to trigger cancer cell apoptosis, but it shows low cytotoxicity in dark. Meanwhile, **TPECM-1TPP** can report the cellular oxidative stress by visualizing the morphological changes of mitochondria. However, **TPECM-2Br** does not target mitochondria and shows no obvious anticancer effect either in dark or under light illumination. This study thus highlights the importance of molecular probe design, which yields a new generation of subcellular targeted molecular theranostic agents with multi-function, such as cancer cell imaging, chemotherapy, photodynamic therapy, and *in situ* monitoring of the therapeutic effect in one go.

## Abstract 2

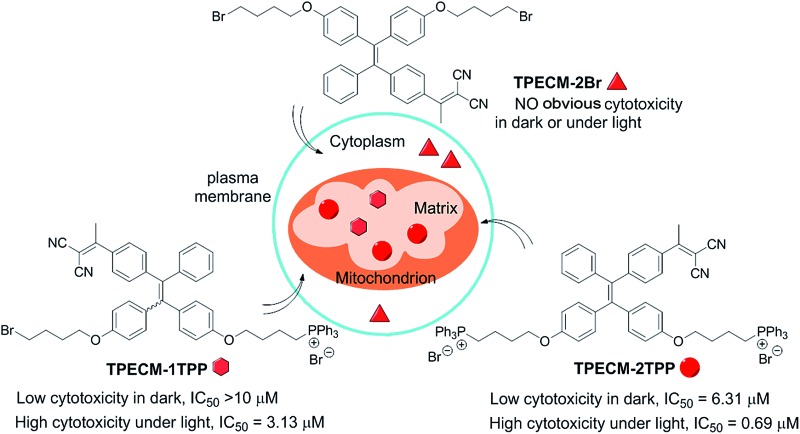
Mitochondria-targeted AIE photosensitizers show multifunctions of targeted and image-guided combination chemotherapy and photodynamic therapy.

## Introduction

Despite significant advances in cancer diagnosis and chemo-therapy, cancer patients continue to suffer from drug resistance, frequent relapses and severe side effects.^1,2^ This highlights the need to develop anticancer agents with new mechanisms of action. Photodynamic therapy (PDT) as a safe, minimally invasive treatment is driven by activating photosensitizers (PSs) to generate reactive oxygen species (ROS), prevalently singlet oxygen for effective cancer cell killing.^3–5^ The combination of PDT and chemotherapy with different therapeutic mechanisms has been proved effective in improving the therapeutic efficiency with minimized side effects, which was achieved mainly *via* two approaches.^6^ The first is to sequentially administrate anticancer drug and PS.^7^ The other is to simultaneously administrate an anticancer drug and a PS which are conjugated together^8–10^ or simply co-encapsulated in nanocarriers.^11,12^ Previous studies have also revealed that the therapeutic efficiency could be further improved when subcellular targeted delivery of therapeutic reagents was achieved as not all the organelles in the cancer cells are equally sensitive to the treatment.^13^ In addition, due to the extremely short half-life (<40 ns) and small radius of action (<20 nm) of singlet oxygen in biological systems,^14,15^ it is expected that direct delivering of PS to specific cells as well as to hypersensitive subcellular sites would greatly enhance the PDT efficiency.

Mitochondria are vital sub-cellular organelles to eukaryotic cells, which play valuable roles in energy production, ROS generation and cellular signaling.^13,16^ Cancer cells often exhibit various degrees of abnormal mitochondrial functions such as change in energy metabolism, higher mitochondrial membrane potential and increased oxidative stress, which provide opportunities to target cancer cell mitochondria for optimal therapeutic efficiency.^17,18^ Several mitochondrial-targeted compounds have been developed as potential anti-cancer agents which either directly influence mitochondria or functionally incur the metabolic alterations in cancer cells with mitochondrial dysfunction.^19–22^ A number of evidences also indicate that the damage of mitochondria is the main reason for PDT-induced cell apoptosis.^23^ Therefore, mitochondrion is the ideal organelle for combined chemotherapy and PDT. To realize mitochondria targeted therapy, the popular strategy is to conjugate mitochondria targeting moiety (*e.g.* mitochondrial localization peptide and lipophilic cation group) to drug/PSs or nanocarriers loaded with therapeutic reagents.^24–27^ It has been reported that lipophilic triphenylphosphonium can help to render several thousand-folds accumulation of its conjugates in mitochondria.^19^ However, as most of the PSs are hydrophobic, they would naturally aggregate in the limited mitochondrial space. The aggregation of PSs will impose a quenched fluorescence emission as well as reduced singlet oxygen generation, which will compromise the quality of imaging and the effect of PDT.^28^

Recently, there is an increasing interest in the development of fluorogens with aggregation induced emission (AIE) characteristics for biological sensing, imaging and cancer therapy applications.^29–36^ The AIE fluorogens (AIEgens) generally have rotor structures, which show very weak fluorescence in molecularly dissolved state but become highly emissive upon aggregation formation.^37^ The optical properties of AIEgens are different from traditional fluorophores, which enabled them to be developed into light-up probes and bright fluorescent nanoparticles for the detection of molecular targets and continuous monitoring of biological processes.^29,38–41^ In addition, several AIEgens were found to show effective ROS generation capabilities for efficient cell ablation.^34,42,43^ We recently also discovered that properly designed AIEgens could also serve as potential potent chemo-drugs for cancer cell killing due to their preferential accumulation in cancer cell mitochondria.^44^ The versatile functions and exciting properties of AIEgens offer the unique opportunity to further develop multifunctional molecular probes for image-guided therapy.

To explore simple molecular probe based image-guided combination chemotherapy and PDT, in this contribution, we developed a series of probes based on a new AIE PS (**TPECM-2Br**). Lipophilic triphenylphosphonium as a mitochondria targeting moiety was selected to conjugate to **TPECM-2Br** because it possesses a delocalized positive charge and can selectively accumulate in cancer cell mitochondria by *trans*-membrane potential gradient.^19^ The obtained **TPECM-1TPP** and **TPECM-2TPP** are almost non-emissive in aqueous media, but they emit strong red fluorescence in aggregated state. The probes showed preferential cellular uptake by cancer cells relative to normal cells, and they can be specifically localized in mitochondria to turn on their fluorescence. **TPECM-2TPP** is found to be able to depolarize mitochondria membrane potential and selectively exert potent chemo-cytotoxicity on the studied cancer cells. Furthermore, the probe can efficiently generate reactive singlet oxygen with strong photo-toxicity upon light illumination, which further enhances the anti-cancer effect. Interestingly, **TPECM-1TPP** only shows high cytotoxicity upon light illumination and has the ability to generate singlet oxygen to cause mitochondrial oxidative stress and trigger cell death. However, **TPECM-2Br** exhibits low cytotoxicity both in dark and upon light illumination in the studied cell lines. These results highlight that molecular design plays an important role in cancer cell treatment.

## Results and discussion

**Scheme 1:**
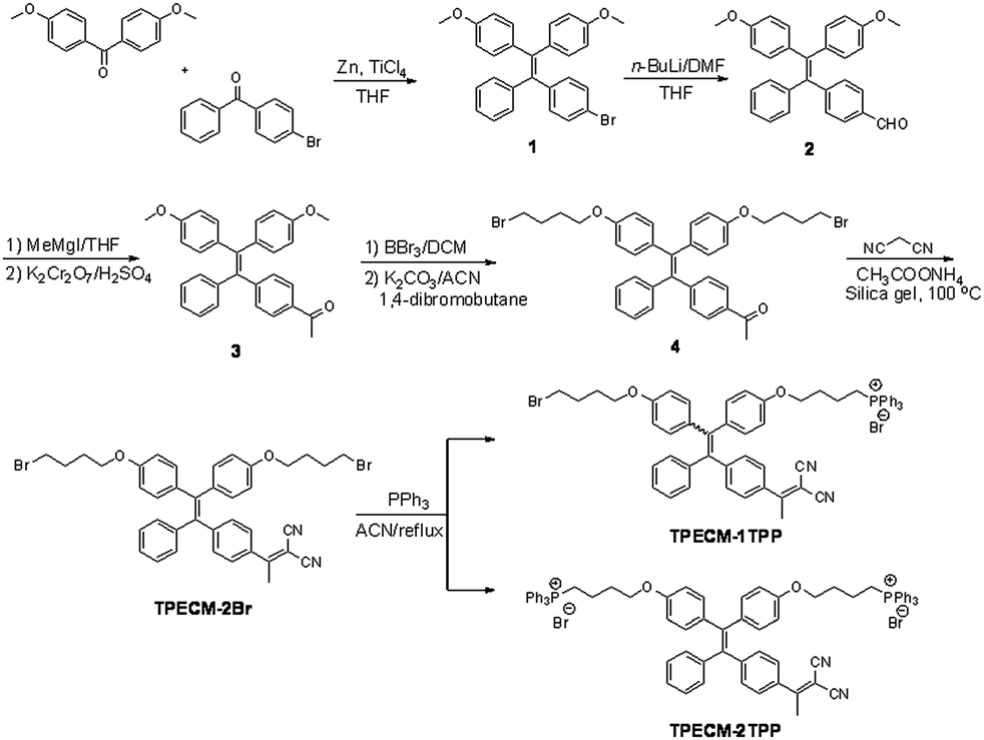
Synthetic route for **TPECM-2Br**, **TPECM-1TPP** and **TPECM-2TPP**.

The probes of **TPECM-2Br**, **TPECM-1TPP** and **TPECM-2TPP** were synthesized according to Scheme 1. Briefly, two different benzophenone derivatives were reacted in the presence of Zn and TiCl_4_ to give **1** in 27.2% yield, which was subsequently treated with *n*-BuLi and DMF to give **2** in 59.7% yield. **2** was first reacted with the Grignard reagent and the resulted secondary alcohol was further oxidized to generate **3** in 61.5% yield. **3** was subsequently treated with boron tribromide, followed by reaction with 4-dibromobutane to give **4** in 13.5% yield. The mixture of **4**, ammonium acetate and malononitrile adsorbed on silica gel was heated at 100 °C for 40 minutes to give **TPECM-2Br** in 74.0% yield, which was then reacted with triphenylphosphine to generate **TPECM-1TPP** in 13.8% yield and **TPECM-2TPP** in 18.2% yield. The purified intermediates and products were well characterized by NMR and mass spectroscopies which confirmed their right structures with high purity (ESI Fig. 1–4†).

**Fig. 1:**
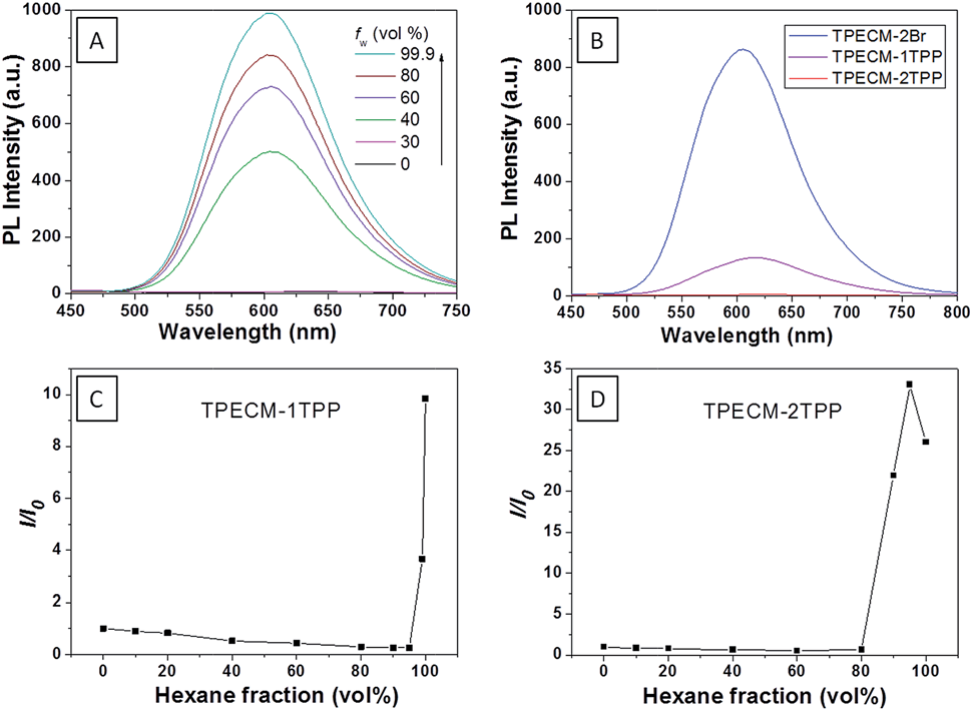
(A) Photoluminescence (PL) spectra of 10 μM **TPECM-2Br** in DMSO–water mixtures with different fractions of water (*f*_w_). (B) Emission spectra of 10 μM **TPECM-2Br**, **TPECM-1TPP** and **TPECM-2TPP** in DMSO–water (v/v = 1/199), respectively. The PL intensity ratios (*I*/*I*_0_) at 628 nm *versus* the solvent composition of the isopropyl alcohol–hexane mixture of 10 μM **TPECM-1TPP** (C) and **TPECM-2TPP** (D). Excitation wavelength: 405 nm. *I*_0_ is the PL intensity of the probes in pure hexane, and *I* is the PL intensity of the same probes in different compositions of isopropyl alcohol and hexane.

We first investigated the photophysical properties of **TPECM-2Br**. **TPECM-2Br** has an absorption maximum at 410 nm in DMSO–water (v/v = 1 : 199) (ESI Fig. 5A†). The photoluminescence (PL) spectra of **TPECM-2Br** were studied in DMSO–water mixtures with different water fractions (*f*_w_). As shown in Fig. 1A, **TPECM-2Br** is faintly fluorescent in DMSO as the compound is well dissolved as molecular species, and the free molecular motion consumes energy, which favors non-radiative decay. However, with gradual increasing *f*_w_, **TPECM-2Br** becomes highly emissive with an emission maximum at 628 nm, showing a characteristic AIE phenomenon. This is due to formation of nanoparticles which activate the radiative decay channel to turn-on the fluorescence. **TPECM-1TPP** and **TPECM-2TPP** in DMSO–water (v/v = 1 : 199) showed similar absorption profiles to that of **TPECM-2Br**. However, their emission spectra in water are very different. As shown in Fig.1B, **TPECM-2Br** is highly emissive in water, while **TPECM-1TPP** shows weak fluorescence and **TPECM-2TPP** is almost non-emissive. To test the AIE characteristics of **TPECM-1TPP** and **TPECM-2TPP**, the mixtures of hexane and isopropyl alcohol were applied to study their fluorescent signals. As shown in Fig. 1C and D, **TPECM-1TPP** and **TPECM-2TPP** become highly emissive when the volume fraction of hexane is gradually increased to more than 80% and the nano-aggregates formation was also confirmed by laser light scattering (LLS) (ESI Fig. 5B–C†). These results indicate that all the three probes are AIE active.

**Fig. 2:**
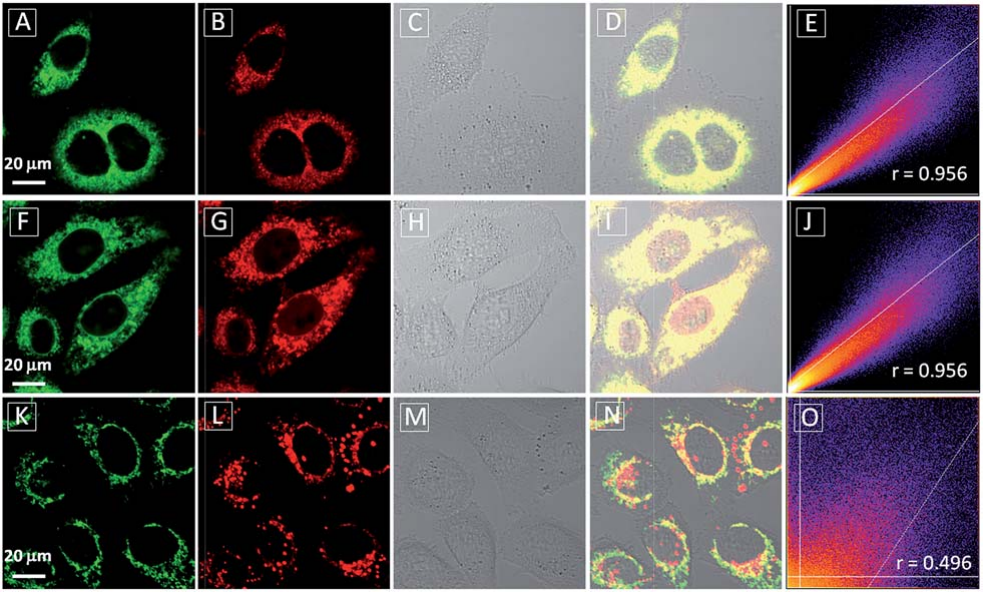
Confocal images of HeLa cells after incubation with 2 μM **TPECM-1TPP** (A–D), **TPECM-2TPP** (F–I) and **TPECM-2Br** (K–N), co-stained with 100 nM Mito-tracker green. The green fluorescence is from Mito-tracker green, *λ*_ex_ = 488 nm and *λ*_em_ = 520 nm ± 20 nm, and the red fluorescence is from the probes, *λ*_ex_ = 405 nm, *λ*_em_ > 560 nm long pass filter. All images share the same scale bar of 20 μm. Co-localization scatter plots for **TPECM-1TPP** (E), **TPECM-2TPP** (J) and **TPECM-2Br** (O) in mitochondria of HeLa cells are also shown for reference.

To assess the intracellular distribution profiles of the probes, HeLa cells were firstly selected due to their well-defined mitochondrial network. As shown in Fig. 2, **TPECM-1TPP** and **TPECM-2TPP** display a characteristic mitochondrial localization pattern, which is consistent with that of the Mito-tracker green, confirming that TPP can effectively drive the accumulation of both probes into mitochondria (Fig. 2A–D and F–I). On the other hand, **TPECM-2Br** is randomly dispersed in the cytoplasm which does not co-localize well with Mito-tracker green (Fig. 2K–N). The Pearson's correlation coefficients are used to quantify the overlaps between the probes and Mito-tracker green, which are 0.96, 0.96 and 0.50 for **TPECM-1TPP**, **TPECM-2PP** and **TPECM-2Br**, respectively (Fig. 2E, J and O).

The effect of probe concentration on the cellular uptake of **TPECM-1TPP** and **TPECM-2TPP** in HeLa cells was also studied. From the confocal images, it is obvious that the higher probe concentration leads to brighter red fluorescence in HeLa cells for both probes (ESI Fig. 6 and 7†). Quantitative analysis of the fluorescence intensity shows that the cellular uptake of **TPECM-1TPP** is ∼25% less efficient than that for **TPECM-2TPP** in HeLa cells under the same probe concentration, which indicates that TPP favors both mitochondria targeting and cellular uptake (ESI Fig. 8†). We then monitored the cellular uptake of 2 μM **TPECM-1TPP** in HeLa, MDA-MB-231 and NIH-3T3 cells at different time points. It was found that for each cell line, the image intensity increases with the probe incubation time from 1 h to 3 h. Quantitative analysis of the fluorescence intensity for the images obtained for cells upon 3 h incubation with the probes reveals that the uptake performance of **TPECM-1TPP** in HeLa and MDA-MB-231 cells is quite similar (ESI Fig. 6 and 9†), which is ∼72% higher than that for NIH-3T3 cells calculated from the results of flow cytometry (ESI Fig. 10–11†). Similarly, **TPECM-2TPP** is also preferably accumulated in the tested cancer cells judging from confocal and flow cytometry studies (ESI Fig. 12–13†). The more accumulation of the probes in the tested cancer cells could be due to the higher mitochondrial membrane potential of cancer cells than that of normal cells.^45^ This potential derivation has been reported to be approximately 60 mV which is sufficient to incur 10-fold greater accumulation of positively charged compounds in cancer cells according to the Nernst equation.^46^

**Fig. 3:**
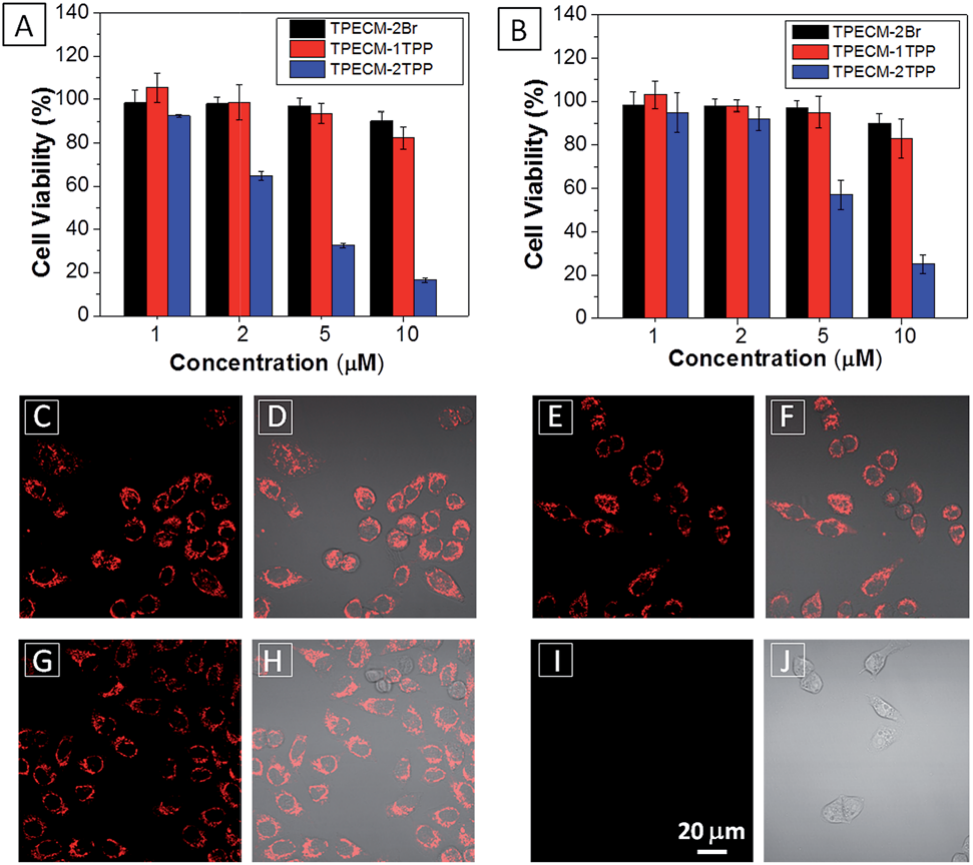
(A) The viability of HeLa cells (A) and NIH-3T3 cells (B) upon treatment with **TPECM-2Br**, **TPECM-1TPP** and **TPECM-2TPP** at different concentrations in dark for 24 h. The mitochondria membrane potential of HeLa cells measured by tetramethyl rhodamine ethyl ester (TMRE) without treatment (C and D), with the treatment of 2 μM **TPECM-2Br** (E and F), 2 μM **TPECM-1TPP** (G and H) and 2 μM **TPECM-2TPP** (I and J) for 3 h. The TMRE was excited at 543 nm and the red fluorescence was detected using a 575–625 nm bandpass filter. All images share the same scale bar of 20 μm.

After confirming that both **TPECM-1TPP** and **TPECM-2TPP** are indeed located in mitochondria, we then tested whether the specific targeting could affect cell viability. MTT assays were used to study cytotoxicity of **TPECM-2Br**, **TPECM-1TPP** and **TPECM-2TPP** under dark. After 24 h incubation, **TPECM-2Br** and **TPECM-1TPP** exhibited low cytotoxicity even at a high concentration of 10 μM as more than 80% of the tested cells survived (Fig. 3A). On the contrary, **TPECM-2TPP** demonstrated much higher dark cytotoxicity with an IC_50_ value of 6.31 μM for HeLa cells. Similar results were also observed for MDA-MA-231 cancer cells, where **TPECM-2Br** and **TPECM-1TPP** exhibited no obvious cytotoxicity but **TPECM-2TPP** showed high dark cytotoxicity with an IC_50_ value of 4.03 μM (ESI Fig. 14 and 15†).

To understand the high dark toxicity for **TPECM-2TPP**, we studied the effect of the probe accumulation in mitochondria on its membrane potential using tetramethylrhodamine ethyl ester (TMRE) as an indicator. TMRE is a fluorescent lipophilic cationic dye which can specifically stain polarized mitochondria with high potential.^47,48^ It is important to note that although both **TPECM-2TPP** and TMRE have red fluorescence, there is no interference signal obtained from the probe upon excitation at 543 nm. In these experiments, HeLa cells were firstly incubated with 2 μM **TPECM-2TPP** for 3 h, which was followed by the co-stain with TMRE and the confocal images are shown in Fig. 3C–J. The TMRE fluorescence disappeared in **TPECM-2TPP** treated HeLa cells (Fig. 3I–J), while bright red fluorescence was observed for the control cells without probe treatment (Fig. 3C and D), or for HeLa cells upon treatment with **TPECM-2Br** (2 μM) or **TPECM-1TPP** (2 μM) under the same conditions (Fig. 3E–H). These results indicate that **TPECM-2TPP** could efficiently depolarize the mitochondrial membrane potential and exert potent cytotoxicity. In addition, strong red fluorescence from TMRE could be detected when **TPECM-2TPP** (2 μM) was incubated with NIH-3T3 (ESI Fig. 16†), implying it induces less mitochondria membrane damage in this case, which agrees with the MTT results (Fig. 3B).

**Fig. 4:**
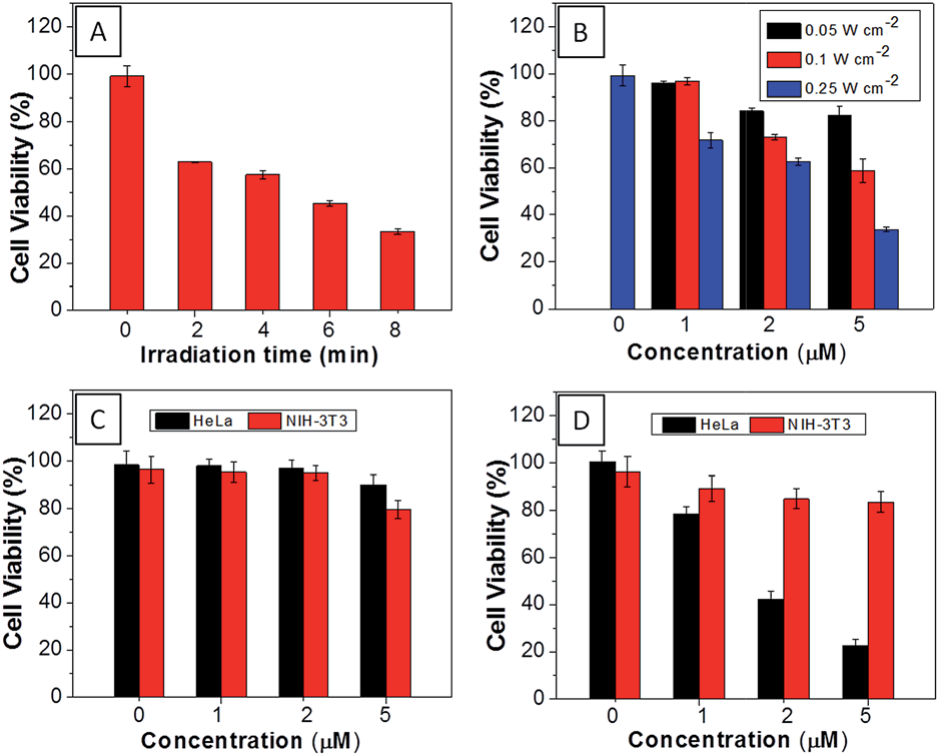
(A) Viability of HeLa cells upon treatment with **TPECM-1TPP** (5 μM) under different duration of white light irradiation (0.25 W cm^–2^). (B) Viability of HeLa cells upon treatment with different concentrations of **TPECM-1TPP** under white light irradiation for 8 min at different powers. The viability of HeLa and NIH-3T3 cells upon treatment with different concentrations of **TPECM-2Br** (C) or **TPECM-1TPP** (D) under white light irradiation (0.25 W cm^–2^, 8 min).

The AIEgen used in this study contained a unique dicyanovinyl group which enabled the probes to serve as PSs to generate singlet oxygen after light irradiation.^34,42^ 9,10-Anthracenediyl-bis(methylene)dimalonic acid (ABDA) is a singlet oxygen indicator, whose absorbance decreases upon interaction with singlet oxygen. When incubating ABDA with **TPECM-2Br** or **TPECM-1TPP** upon white light irradiation (ESI Fig. 17†), the absorbance of ABDA decreases quickly, indicating that both **TPECM-2Br** and **TPECM-1TPP** could efficiently generate singlet oxygen. However, the two probes show very different photo-toxicity towards HeLa cells under white light irradiation. As shown in Fig. 4, **TPECM-2Br** has no obvious toxicity even at a high concentration of 10 μM. However, **TPECM-1TPP** exhibits time- and power-dependent photo-toxicity, which can kill more than 70% of HeLa cells at 5 μM, with an IC_50_ value of 3.13 μM upon white light illumination. The difference in photo-toxicity is largely because mitochondria are more prone to singlet oxygen attack than cytoplasm.^49^ In addition, **TPECM-1TPP** does not affect viability of NIH-3T3 cells as over 90% of cells survived after the treatment (Fig. 4D). As a consequence, **TPECM-1TPP** could serve as a good PS because of its low dark cytotoxicity, high photo-toxicity and selective targeting ability towards the tested cancer cells.

**Fig. 5:**
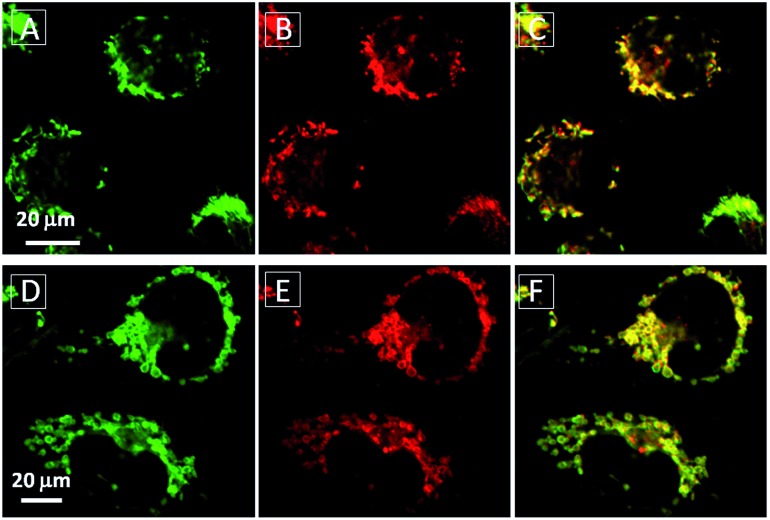
The mitochondrial morphology change of MDA-MB-231 cells after treatment with **TPECM-1TPP** (5 μM) under dark (A–C) or light irradiation (0.1 W cm^–2^, 8 min) (D–F). A and D are images from Mito-tracker green, *λ*_ex_ = 488 nm; *λ*_em_ = 520 nm ± 20 nm. B and E are images from **TPECM-1TPP**, *λ*_ex_ = 405 nm; *λ*_ex_ > 560 nm long pass filter. C and F are overlay images from Mito-tracker green and **TPECM-1TPP**.

Additionally, **TPECM-1TPP** was also found to be able to visualize the mitochondria morphological changes under high oxidative stress induced by light-irradiation (ESI Fig 18†). As shown in Fig. 5, under the dark condition, mitochondria in **TPECM-1TPP**-treated cells were tubular-like. But after white light irradiation, mitochondria adopted small round shapes. The swelling of mitochondria is another evidence to indicate the depolarization of the mitochondrial membrane potential.^50^ As such, **TPECM-1TPP** is not only a good PS, but also an imaging tool to monitor the mitochondria morphological change during PDT.

**Fig. 6:**
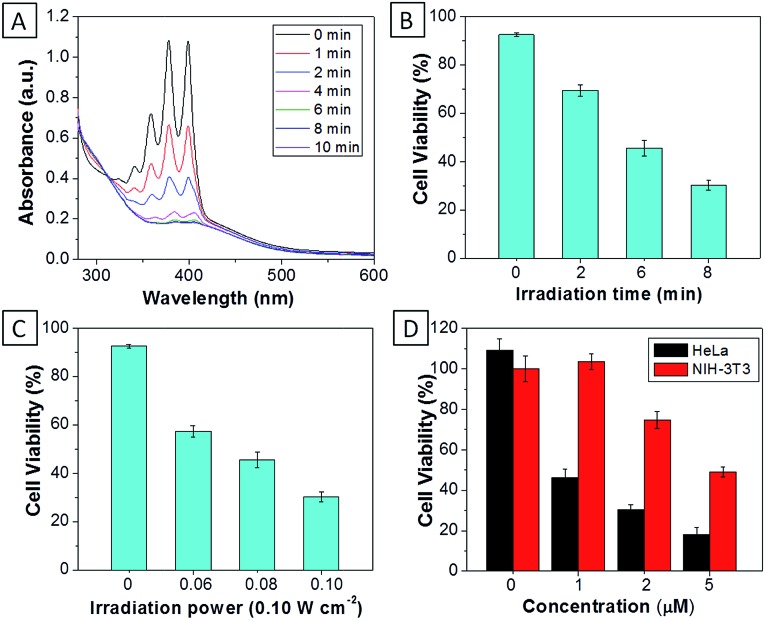
(A) The absorption spectra of ABDA (100 μM) in the presence of **TPECM-2TPP** (20 μM) after different durations of white light irradiation. The viability of HeLa cells upon treatment with **TPECM-2TPP** (5 μM) under different durations of white light irradiation (0.10 W cm^–2^) (B), or under white light irradiation for 8 min at different power (C). (D) The viability of HeLa and NIH-3T3 cells upon treatment with different concentrations of **TPECM-2TPP** under white light irradiation (0.10 W cm^–2^, 8 min).

For **TPECM-2TPP**, in addition to the high cytotoxicity under dark conditions, under light illumination, it could also generate singlet oxygen to bleach the indicator of ABDA. As shown in Fig. 6A, it took 20 μM **TPECM-2TPP** less than 6 min to almost completely bleach 100 μM ABDA under light irradiation. When the probe was used for cell based assays, the viabilities were studied for HeLa cells upon treatment with the probe under different durations of white light irradiation at a constant power of 0.10 W cm^–2^ (Fig. 6B) or under white light irradiation at different powers for a fixed period of time (Fig. 6C). At the same concentration of the probe, **TPECM-2TPP** showed stronger inhibition of cell viability with longer irradiation time at higher irradiation power. The high cytotoxicity and the singlet oxygen generation capability thus offered a new opportunity for **TPECM-2TPP** to be used as a unique molecular probe for combined chemotherapy and PDT. Notably, under white light illumination, the IC_50_ value of the probe towards HeLa cells is 0.69 μM (ESI Fig. 15†), which is 8-fold lower than that of the probe without light irradiation (6.31 μM). The light-induced increase in cell killing efficiency of the probe was also observed in another cancer cell line (MDA-MB-231) where the probe showed an IC_50_ value of 2.48 μM under light illumination which compares favorably to that obtained in the dark (4.03 μM). It is also important to note that **TPECM-2TPP** shows much less phototoxicity to NIH-3T3 than that for HeLa cells (Fig. 6D), which is consistent with the observation that NIH-3T3 cells uptake less amount of the probe than HeLa cells.

**Fig. 7:**
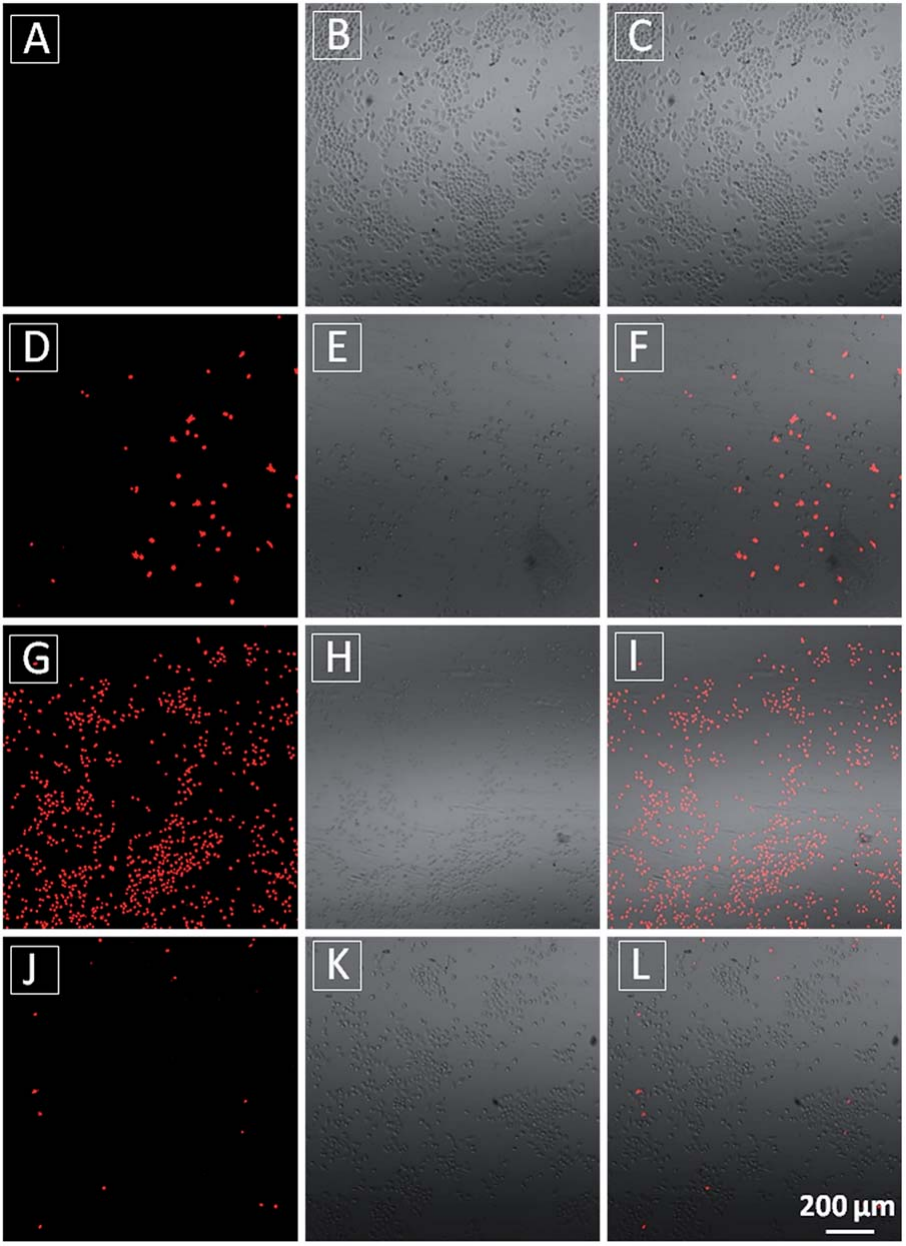
Confocal fluorescence (A, D, G and J), bright field (B, E, H and K) and overlay fluorescence and bright field (C, F, I and L) images of PI stained HeLa cells after incubation of the cells without **TPECM-2TPP** (A–C), or with **TPECM-2TPP** (1 μM) in dark for 24 h (D–F) or with **TPECM-2TPP** (1 μM) for 3 h in dark followed by washing-away of the probe, white light irradiation (8 min, 0.10 W cm^–2^) and further incubation for 24 h (G–I) or with **TPECM-2TPP** (1 μM) for 3 h in dark followed by washing-away of the probe, pre-incubation with Vitamin C (100 μM, 15 min), white light irradiation (8 min, 0.10 W cm^–2^) and further incubation for 24 h (J–L).

The MTT results were further confirmed by propidium iodide (PI) staining. PI, a cell impermeable dye, only stains dead cells or late apoptotic cells with damaged membrane. As shown in Fig. 7, only part of **TPECM-2TPP**-treated cells incubated under the dark was stained whereas nearly all the cells were stained after they were exposed to white light irradiation (8 min, 0.10 W cm^–2^). Meanwhile, when the cells were treated with both the probe and singlet oxygen scavenger (Vitamin C), much fewer cells were stained even after white light irradiation, indicating that the singlet oxygen generation plays an important role in cell killing. Collectively, these results indicate that the combination chemo-therapy and PDT provides higher anticancer effect compared to the single therapeutic approach alone.

## Conclusion

In summary, we have systematically designed and synthesized three molecular probes with aggregation-induced emission characteristics. Without the targeting group, **TPECM-2Br** is randomly dispersed in cytoplasm and renders no obvious cytotoxicity in dark or under white light irradiation. By virtue of the subcellular targeting TPP group, both **TPECM-1TPP** and **TPECM-2TPP** are able to specifically aggregate and light up in mitochondria. With one TPP group, **TPECM-1TPP** shows low cytotoxicity to all the tested cells even at a concentration of 10 μM. However, it shows much higher cytotoxicity (IC_50_ = 3.13 μM) under light irradiation. In addition, **TPECM-1TPP** is able to monitor the morphological change of mitochondria, and its phototoxicity is more potent for HeLa and MDA-MB-231 cells than NIH-3T3 cells. The low dark cytotoxicity, high photo-cytotoxicity and preferable accumulation in the tested cancer cells makes **TPECM-1TPP** a good AIE PS. Different from **TPECM-1TPP**, with two TPP groups, **TPECM-2TPP** is able to specifically depolarize mitochondria membrane potential of HeLa cells and exert strong chemo-cytotoxicity in dark. It also efficiently generates singlet oxygen and induces photo-cytotoxicity under white light irradiation to yield an overall IC_50_ of 0.69 μM for HeLa cells. The distinct performance among the three probes highlights the importance of molecular probe design in realizing targeted therapy in mitochondria. In addition, as compared to the existing systems for combined chemo- and photodynamic therapy, which generally require the conjugation between drug and PS or co-encapsulating them into nanoparticles, **TPECM-2TPP** represents the first molecular probe for image-guided combination chemotherapy and PDT without direct drug conjugation. The probe of **TPECM-2TPP** thus represents a new generation of subcellular targeted theranostic agent with multifunction, such as cancer cell detection, imaging, chemotherapy, and photodynamic therapy. The concept and simplicity in our probe design thus provides the basis for future design of molecule probes for targeted and image-guided combination therapy.

## References

[cit1] Umar A., Dunn B. K., Greenwald P. (2012). Nat. Rev. Cancer.

[cit2] Kelland L. (2007). Nat. Rev. Cancer.

[cit3] Secret E., Maynadier M., Gallud A., Gary-Bobo M., Chaix A., Belamie E., Maillard P., Sailor M. J., Garcia M., Durand J.-O., Cunin F. (2013). Chem. Commun..

[cit4] Celli J. P., Spring B. Q., Rizvi I., Evans C. L., Samkoe K. S., Verma S., Pogue B. W., Hasan T. (2010). Chem. Rev..

[cit5] Agostinis P., Berg K. (2011). Ca-Cancer J. Clin..

[cit6] Olivo M., Bhuvaneswari R., Lucky S. S., Dendukuri N., Soo-Ping Thong P. (2010). Pharmaceuticals.

[cit7] Zuluaga M.-F., Lange N. (2008). Curr. Med. Chem..

[cit8] Lau J. T. F., Lo P.-C., Fong W.-P., Ng D. K. P. (2012). J. Med. Chem..

[cit9] Lottner C., Knuechel R., Bernhardt G., Brunner H. (2004). Cancer Lett..

[cit10] Králová J., Kejík Z., Bríza T., Poucková P., Král A., Martásek P., Král V. (2010). J. Med. Chem..

[cit11] Khdair A., Chen D., Patil Y., Ma L., Dou Q. P., Shekhar M. P. V., Panyam J. (2010). J. Controlled Release.

[cit12] Khdair A., Handa H., Mao G., Panyam J. (2009). Eur. J. Pharm. Biopharm..

[cit13] Rajendran L., Knölker H.-J., Simons K. (2010). Nat. Rev. Drug Discovery.

[cit14] Juarranz Á., Jaén P., Sanz-Rodríguez F., Cuevas J., González S. (2008). Clin. Transl. Oncol..

[cit15] Ogilby P. R. (2010). Chem. Soc. Rev..

[cit16] Wang X., Peralta S. and Moraes C. T., Mitochondrial alterations during carcinogenesis: a review of metabolic transformation and targets for anticancer treatments., Elsevier Inc., 1st edn, vol. 119, 2013. 10.1016/B978-0-12-407190-2.00004-6 23870511

[cit17] Galluzzi L., Larochette N., Zamzami N., Kroemer G. (2006). Oncogene.

[cit18] Byoung H. K., Plescia J., Ho Y. S., Meli M., Colombo G., Beebe K., Scroggins B., Neckers L., Altieri D. C. (2009). J. Clin. Invest..

[cit19] Smith R. a J., Hartley R. C., Murphy M. P. (2011). Antioxid. Redox Signaling.

[cit20] Wang F., Ogasawara M. A., Huang P. (2010). Mol. Aspects Med..

[cit21] Jean S. R., Tulumello D. V., Wisnovsky S. P., Lei E. K., Pereira M. P., Kelley S. O. (2014). ACS Chem. Biol..

[cit22] Fulda L., Galluzzi L., Kroemer G. (2010). Nat. Rev. Drug Discovery.

[cit23] Hilf R. (2007). J. Bioenerg. Biomembr..

[cit24] Sakhrani N. M., Padh H. (2013). Drug Des., Dev. Ther..

[cit25] Li S. P.-Y., Lau C. T.-S., Louie M.-W., Lam Y.-W., Cheng S. H., Lo K. K.-W. (2013). Biomaterials.

[cit26] Prasad P., Khan I., Kondaiah P., Chakravarty A. R. (2013). Chem.–Eur. J..

[cit27] Xu J., Zeng F., Wu H., Hu C., Wu S. (2014). Biomacromolecules.

[cit28] Rajaputra P., Nkepang G., Watley R., You Y. (2013). Bioorg. Med. Chem..

[cit29] Shi H., Kwok R. T. K., Liu J., Xing B., Tang B. Z., Liu B. (2012). J. Am. Chem. Soc..

[cit30] Samanta S., Goswami S., Hoque M. N., Ramesh A., Das G. (2014). Chem. Commun..

[cit31] Li X., Ma K., Zhu S., Yao S., Liu Z., Xu B., Yang B., Tian W. (2014). Anal. Chem..

[cit32] Li Y., Yu H., Qian Y., Hu J., Liu S. (2014). Adv. Mater..

[cit33] Zhao E., Deng H., Chen S., Hong Y., Leung C. W. T., Lam J. W. Y., Tang B. Z. (2014). Chem. Commun..

[cit34] Hu F., Huang Y., Zhang G., Zhao R., Yang H., Zhang D. (2014). Anal. Chem..

[cit35] Yuan Y., Kwok R. T. K., Tang B. Z., Liu B. (2014). J. Am. Chem. Soc..

[cit36] Kwok R. T. K., Leung C. W. T., Lam J. W. Y., Tang B. Z. (2015). Chem. Soc. Rev..

[cit37] Mei J., Hong Y., Lam J. W. Y., Qin A., Tang Y., Tang B. Z. (2014). Adv. Mater..

[cit38] Ding D., Li K., Liu B., Tang B. Z. (2013). Acc. Chem. Res..

[cit39] Zhang L., He N., Lu C. (2015). Anal. Chem..

[cit40] Liang J., Tang B. Z., Liu B. (2015). Chem. Soc. Rev..

[cit41] Li K., Liu B. (2014). Chem. Soc. Rev..

[cit42] Yuan Y., Zhang C.-J., Gao M., Zhang R., Tang B. Z., Liu B. (2015). Angew. Chem., Int. Ed..

[cit43] Yuan Y., Feng G., Qin W., Tang B. Z., Liu B. (2014). Chem. Commun..

[cit44] Hu Q., Gao M., Feng G., Liu B. (2014). Angew. Chem., Int. Ed..

[cit45] Modica-Napolitano J. S., Aprille J. R. (2001). Adv. Drug Delivery Rev..

[cit46] Modica-Napolitano J. S., Aprille J. R. (1987). Cancer Res..

[cit47] Chazotte B. (2011). Cold Spring Harb Protoc..

[cit48] Perry S. W., Norman J. P., Barbieri J., Brown E. B., Gelbard H. A. (2011). BioTechniques.

[cit49] Qi Y. B., Garren E. J., Shu X., Tsien R. Y., Jin Y. (2012). Proc. Natl. Acad. Sci. U. S. A..

[cit50] Ly J. D., Grubb D. R., Lawen A. (2003). Apoptosis.

