# Supplementary material for: Image-guided combination chemotherapy and photodynamic therapy using a mitochondria-targeted molecular probe with aggregation-induced emission characteristics
Source: Chem Sci. 2015 May 18;6(8):4580–6. doi: 10.1039/c5sc00826c (PMC5500860; doi:10.1039/c5sc00826c)
Supplement: Supplementary file 1 [file SC-006-C5SC00826C-s001.pdf]

Electronic Supplementary Material (ESI) for Chemical Science.
This journal is © The Royal Society of Chemistry 2015

*Supporting Information*

# Image-Guided Combination Chemotherapy and Photodynamic Therapy Using a Mitochondria-Targeted Molecular Probe with Aggregation-Induced Emission Characteristics

Chong-jing Zhang,[†,‡] Qinglian Hu,[†,‡] Guangxue Feng,[†] Ruoyu Zhang,[†] Youyong Yuan[†], Xianmao Lu[†], Bin Liu[*,†,§]

[†]Department of Chemical and Biomolecular Engineering, National University of Singapore, 4 Engineering Drive 4, Singapore, 117585

[§]Institute of Materials Research and Engineering, Agency for Science, Technology and Research, 3 Research Link, Singapore, 117602

* Corresponding author (E-mail: cheliub@nus.edu.sg)

## Experimental Section

**General Information** 9,10-Anthracenediyl-bis(methylene)dimalonic acid (ABDA), 3-(4,5-dimethyl-thiazol-2-yl)-2,5-diphenyltetrazolium bromide (MTT), 2′,7′-dichlorodihydrofluorescein diacetate (DCF-DA) and other chemicals were all purchased from Sigma-Aldrich and used as received without further purification. Tetrahydrofuran (THF) was dried through distillation using sodium as drying agent and benzophenone as indicator and used immediately. Dry dichloromethane and dimethylformamide (DMF) were distilled over $CaH_2$. All non-aqueous reactions were carried out in oven-dried glassware under nitrogen atmosphere.

**Synthesis of compounds 1, 2 and 3**

The synthesis of compounds **1**, **2** and **3** followed the previously reported procedure.[1] In the new

batch of synthesis, bis(4-methoxyphenyl)methanone (7.0 g) and (4-bromophenyl)(phenyl) methanone (8.0 g) were reacted to yield compound **1** as white solid (3.1 g, 27.2% yield); compound **1** (1.5 g) was reacted with n-butyllithium and DMF to yield compound **2** as yellow green solid (800 mg, 59.7% yield); compound **2** was treated with methyl magnesium iodide and potassium dichromate to yield compound **3** as light green solid (508 mg, 61.5% yield).

**Synthesis of compound 4**

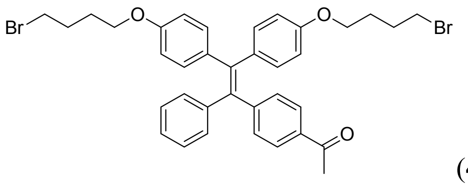


(**4**)

To the solution of compound **3** (95 mg, 0.22 mmol) in dichloromethane (5 mL) was added boron tribromide (1 M, 0.8 mL) in an ice-water bath. Then the reaction was stirred at room temperature for 2 h. The reaction was quenched by addition of water (10 mL) and dichloromethane (20 mL) in an ice-water bath. The organic layer was separated, washed with water (30 mL × 3), brine (30 mL) and dried over $MgSO_4$. The mixture was filtered and the resulting filtrate was concentrated to give a brown residue. This residue was then dissolved in acetonitrile (5 mL), followed by addition of 1,4-dibromobutane (130 mg, 0.60 mmol) and potassium carbonate (41 mg, 0.3 mmol). The resulting mixture was stirred at 60 $^{o}$C for 27 h. After the reaction was cooled down to room temperature, silica gel was added, and the solvent was removed under reduced pressure. The desired solid was purified with chromatography (hexane/ethyl acetate (v/v) = 20/1) to yield **2** as a light green solid (20 mg, 13.5% yield). $^{1}$H NMR (500 MHz, $CDCl_3$) δ 7.70 (d, $J$ = 8.5 Hz, 2H), 7.12 (m, 5 H), 7.00 (dd, $J_1$ = 1.5 Hz, $J_2$ = 7.5 Hz, 2H), 6.92 (dd, $J_1$ = 1.5 Hz, $J_2$ = 8.5 Hz, 4H), 6.63 (dd, $J_1$ = 2.0 Hz, $J_2$ = 8.5 Hz, 4H), 3.93 (dt, $J_1$ = 2.0 Hz, $J_2$ = 6.0 Hz, 4H), 3.48 (dt, $J_1$ = 3.0 Hz, $J_2$ = 7.0 Hz, 4H), 2.52 (s, 3H), 2.02-2.06 (m, 4H), 1.88-1.92 (m, 4H); $^{13}$C NMR (125 MHz, $CDCl_3$) δ

197.7, 157.7, 157.6, 149.7, 143.6, 141.8, 138.1, 135.9, 135.8, 134.6, 132.63, 132.60, 131.5, 131.3, 127.9, 127.8, 126.4, 113.7, 113.5, 66.6, 33.4, 29.4, 27.8, 26.5, 14.0; MS (EI$^+$) m/z: 674.30 (Calcd for $[M]^+$: 674.10).

**Synthesis of TPECM-2Br**

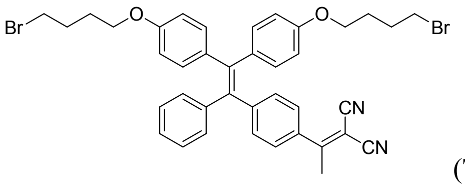


(**TPECM-2Br**)

Compound **4** (20 mg, 0.03 mmol), malononitrile (21 mg, 0.32 mmol) and ammonium acetate (36 mg, 0.46 mmol) were dissolved in the mixture of dichloromethane (5 mL) and methanol (1 mL). Then silica gel (404 mg) was added to the above mixture, and the solvent was removed under reduced pressure. The resulting mixture was heated at 100 $^o$C for 40 minutes. The mixture was cooled down and subsequently separated with chromatography (hexane/ethyl acetate (v/v) = 20/1) to give the desired product as orange solid (16 mg, 74.0% yield). $^1$H NMR (500 MHz, $CDCl_3$) δ 7.33 (d, $J$ = 8.5 Hz, 2H), 7.14 (m, 5H), 7.01 (m, 2H), 6.92 (dd, $J_1$ = 3.0 Hz, $J_2$ = 8.5 Hz, 4H), 6.64 (d, $J$ = 8.5 Hz, 2H), 6.62 (d, $J$ = 9.0 Hz, 2H), 3.93 (q, $J$ = 6.0 Hz, 4H), 3.48 (dt, $J_1$ = 3.0 Hz, $J_2$ = 7.0 Hz, 4H), 2.57 (s, 3H), 2.01-2.05 (m, 4H), 1.89-1.91 (m, 4H); $^{13}$C NMR (125 MHz, $CDCl_3$) δ 174.4, 157.9, 157.7, 149.2, 143.3, 142.5, 137.5, 135.6, 135.4, 133.0, 132.7, 132.6, 131.8, 131.3, 128.0, 126.9, 126.5, 113.8, 113.5, 70.5, 66.7, 66.6, 33.5, 33.4, 29.4, 27.8, 23.8; HRMS (ESI$^-$) m/z: 721.1046 (Calcd for $[M-H]^-$: 721.1071).

**Synthesis of TPECM-1TPP and TPECM-2TPP**

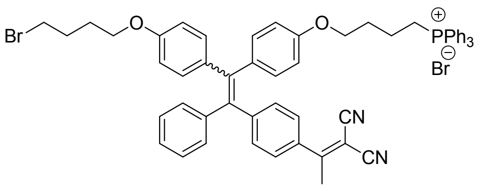


(**TPECM-1TPP**)

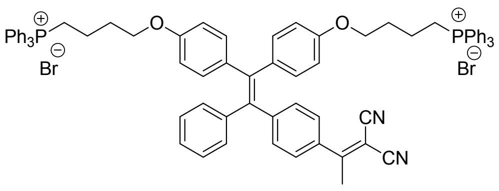


(**TPECM-2TPP**)

To the solution of compound **TPECM-2Br** (16 mg, 0.022 mmol) in acetonitrile (5 mL) was added triphenylphosphine (64 mg, 0.24 mmol). The resulting mixture was refluxed for 48 hours. Then the solvent was removed under reduced pressure. The residue was washed with hexane (10 mL) and the remaining residue was purified with HPLC to give the product **TPECM-1TPP** (3 mg, orange oil), $^{1}$H NMR (500 MHz, Methanol-$d_4$) δ 7.90 (q, $J$ = 7.0 Hz, 3H), 7.81-7.71 (m, 12H), 7.42 (d, $J$ = 8.5 Hz, 1H), 7.36 (d, $J$ = 8.5 Hz, 1H), 7.14 (m, 5H), 7.01 (d, $J$ = 7.0 Hz, 1H), 6.89-6.93 (m, 4H), 6.60-6.68 (m, 4H), 4.00 (q, $J$ = 5.5 Hz, 2H), 3.94 (m, 2H), 3.51 (q, $J$ = 7.0 Hz, 2H), 3.44 (m, 2H), 2.58 (s, 1.5H), 2.55 (s, 1.5H), 1.97-2.03 (m, 4H), 1.88 (m, 4H); HRMS (ESI) m/z: 905.2900 (Calcd for $[M\text{-}Br]^{+}$: 905.2866); and **TPECM-2TPP** (5 mg, orange oil), $^{1}$H NMR (500 MHz, DMSO-$d_6$) δ 7.90 (t, $J$ = 7.5 Hz, 6H), 7.80-7.71 (m, 24H), 7.47 (d, $J$ = 8.5 Hz, 6H), 7.06-7.17 (m, 3H), 7.07 (d, $J$ = 8.0 Hz, 2H), 6.98 (d, $J$ = 7.0 Hz, 2H), 6.86 (dd, $J_1$ = 2.5 Hz, $J_2$ = 8.5 Hz, 4H), 6.65 (d, $J$ = 8.0 Hz, 4H), 3.95-3.90 (m, 4H), 2.53 (s, 3H), 1.86 (m, 4H), 1.66 (m, 4H); HRMS (ESI) m/z: 544.2281 (Calcd for $[M\text{-}2Br]^{2+}$: 544.2294).

**Cell culture**

HeLa human cervix carcinoma cells, MDA-MB-231 human breast cancer cells, NIH-3T3 mouse embryonic fibroblast cells were provided by American Type Culture Collection (ATCC). The cells were cultured in DMEM (Invitrogen, Carlsbad, CA) containing 10% heat-inactivated FBS (Invitrogen), 100 U mL$^{-1}$ penicillin, and 100 μg mL$^{-1}$ streptomycin (Thermo Scientific) and were

maintained in a humidified incubator at 37 °C with 5% $CO_2$. Before experiments, the cells were pre-cultured until confluence was reached.

**Intracellular localization of TPECM-2Br, TPECM-1TPP and TPECM-2TPP**

HeLa cells were cultured in the chambers (LAB-TEK, Chambered Coverglass System) at a density of $5\times10^5$ per mL for 18 h. The culture medium was removed, and the cells were rinsed with PBS. HeLa cells were incubated with **TPECM-2Br** (2 μM), **TPECM-1TPP** (1, 2 and 5 μM), **TPECM-2TPP** (1, 2 and 5 μM) at 37 °C for 3 h. For co-localization study, cells were washed with PBS, 200 nM of Mito-Tracker green was added and incubated at 37 °C for 45 min. After washing with PBS for 3 times, cells were placed on ice and imaged by confocal laser scanning microscope (CLSM, Zeiss LSM 410, Jena, Germany). For **TPECM-2Br**, **TPECM-1TPP** and **TPECM-2TPP**, the excitation was 405 nm, and the band filter was 560 nm; for Mito-Tracker imaging, the excitation was 488 nm, and the emission filter was 510-560 nm.

To study photo-induced mitochondria morphology change, the MDA-MB-231 cells were cultured in the chamber at a density of $5\times10^5$ per mL for 18 h. After incubation with 5 μM of **TPECM-1TPP** for 3 h in the dark, the cells were irradiated for 8 min at the power density of 0.25 W $cm^{-2}$. Then the cells were stained with 200 nM Mito-Tracker green at 37 °C for 45 min and immediately imaged by confocal laser scanning microscope (CLSM, Zeiss LSM 410, Jena, Germany).

**Cellular uptake assay**

HeLa, MDA-MB-231 and NIH-3T3 cells were seeded onto 6-well plates and grown for 18 h to achieve 80% confluence at a density of $1\times10^6$ cells per well. To compare cellular uptake among different cell lines, the cells were incubated with **TPECM-1TPP** (3 μM) or **TPECM-2TPP** (3 μM) at 37°C for 2 h. The cells without treatment were used as control. Then the cells were washed

with PBS, trypsinized, and re-suspended in PBS. Flow cytometry measurements were conducted using Cyan-LX (Dako Cytomation) with excitation at 405 nm and emission above 605 nm. The mean fluorescence was determined by counting 10,000 events.

**Cell cytotoxicity assay**

The cytotoxicity of the compounds was evaluated using the MTT assay. The HeLa, MDA-MB-231 and NIH-3T3 cells were seeded in 96-well plates at a density of 5000 cells/well. After incubation for 24 h, the medium was replaced with freshly prepared solution of **TPECM-2Br**, **TPECM-1TPP** and **TPECM-2TPP** at different concentrations and further incubated at 37 °C for 3 h in the dark. Fresh medium was added and further incubated for 24 h. For the phototoxicity assay using **TPECM-2Br** and **TPECM-1TPP**, after incubation with **TPECM-2Br** and **TPECM-1TPP** at different concentrations in the dark for 3 h, the cells were irradiated with white light for 8 min at the power of 0.05, 0.1 and 0.25 W $cm^{-2}$, respectively. For phototoxicity assay with the different irradiation time, the cells were irradiated for 2, 4, 6 and 8 min at the power of 0.25 W $cm^{-2}$. For phototoxicity assays using **TPECM-2TPP**, after incubation of the cells with the probes at different concentrations in the dark for 3 h, the cells were irradiated with white light for 8 min at the power of 0.06, 0.08 and 0.10 W $cm^{-2}$, respectively. For phototoxicity assays with different irradiation time, the cells were irradiated for 2, 6 and 8 min at the power of 0.10 W $cm^{-2}$. After light irradiation, the cell medium was replaced with fresh medium and further incubated for 24 h. After washing the cells with PBS buffer, 100 μL of freshly prepared MTT (0.5 mg $mL^{-1}$) solution in PBS was added into each well and kept at 37 °C for 3 h. After MTT medium removal, the formazan crystals were dissolved in DMSO and the absorbance was measured at 570 nm using a microplate reader (Genios Tecan). The untreated cells were served as the control and their

viability was set as 100%.

**Intracellular ROS generation**

2’,7’-Dichlorofluorescein diacetate (DCF-DA) was used to detect the generation of cellular reactive oxygen species (ROS). Once the cell-permeable DCF-DA was oxidized by cellular ROS, it generated a fluorescent compound DCF with an emission maximum at 528 nm. HeLa cells were cultured in the chambers (LAB-TEK, Chambered Coverglass System) at 37 °C. After 80% confluence, the culture medium was removed and washed twice with PBS buffer. Following incubation with **TPECM-1TPP** (5 μM) for 3 h in dark, DCF-DA was loaded into the cells. After 5 min incubation, cells were washed twice with PBS and then exposed to light irradiation for 1 min at the power density of 0.25 W $cm^{-2}$. For **TPECM-1TPP**, the excitation was 405 nm, and the band filter was 560 nm; for DCF-DA detection, the excitation was 488 nm, and the emission filter was 510-560 nm.

**Cell apoptosis detection**

HeLa cells were cultured in the chambers (LAB-TEK, Chambered Coverglass System) at a density of $5\times10^5$ per mL for 18 h. The culture medium was removed, and the cells were rinsed with PBS. Following by incubation of the cells with **TPECM-2TPP** (1 μM) at 37 °C for 3 h in the dark, the medium was removed and the cells were washed twice with PBS. For pretreatment with Vitamin C, the cells were further incubated with Vitamin C (100 μM) for 15 min. For the phototoxicity study, the cells were exposed to light irradiation for 8 min at the power density of 0.10 W $cm^{-2}$ and further incubated for 24 h. For the dark control, the cells were treated with **TPECM-2TPP** (1 μM) for 24 h. The cellular apoptosis imaging measurement was carried out based on standard detection kit (PI stain) according to manufacturer’s protocol. The cells were

then imaged immediately by confocal laser scanning microscope (CLSM, Zeiss, LSM 410, Jena, Germany).

References

1 Y. Yuan, C.-J. Zhang, M. Gao, R. Zhang, B. Z. Tang and B. Liu, *Angew. Chem. Int. Ed.*, 2015, **54**, 1780–6.

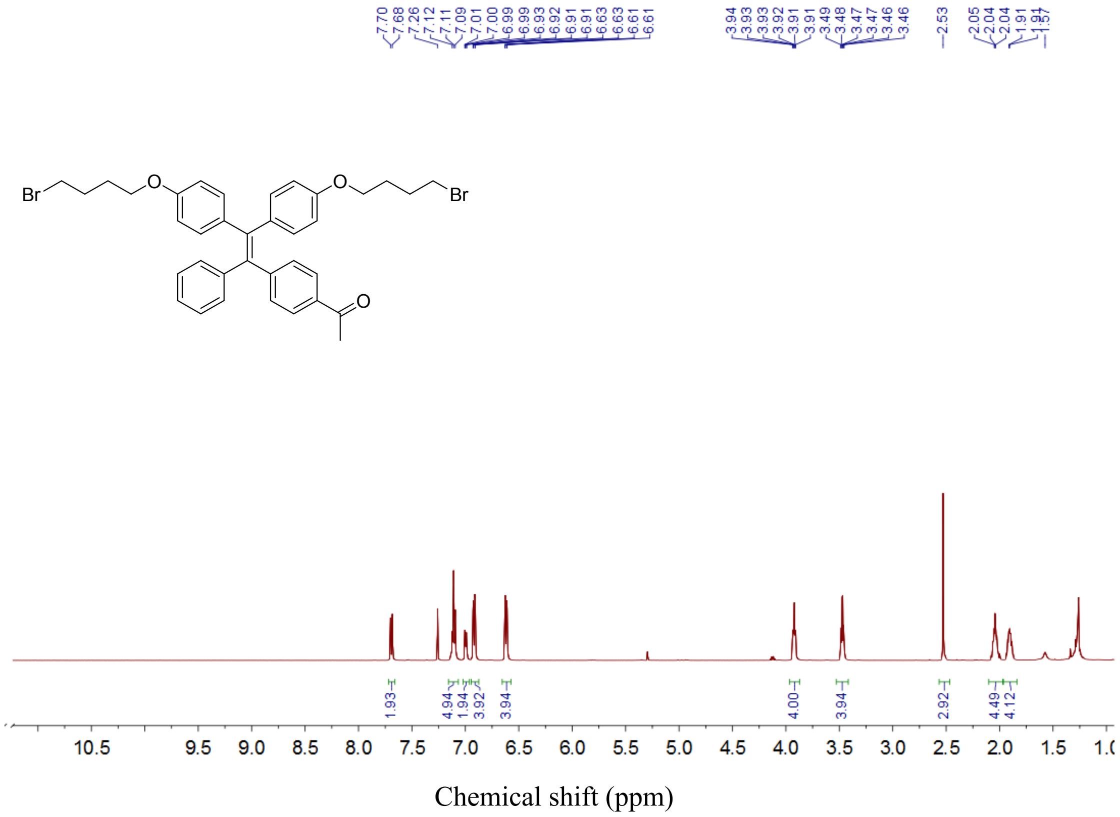

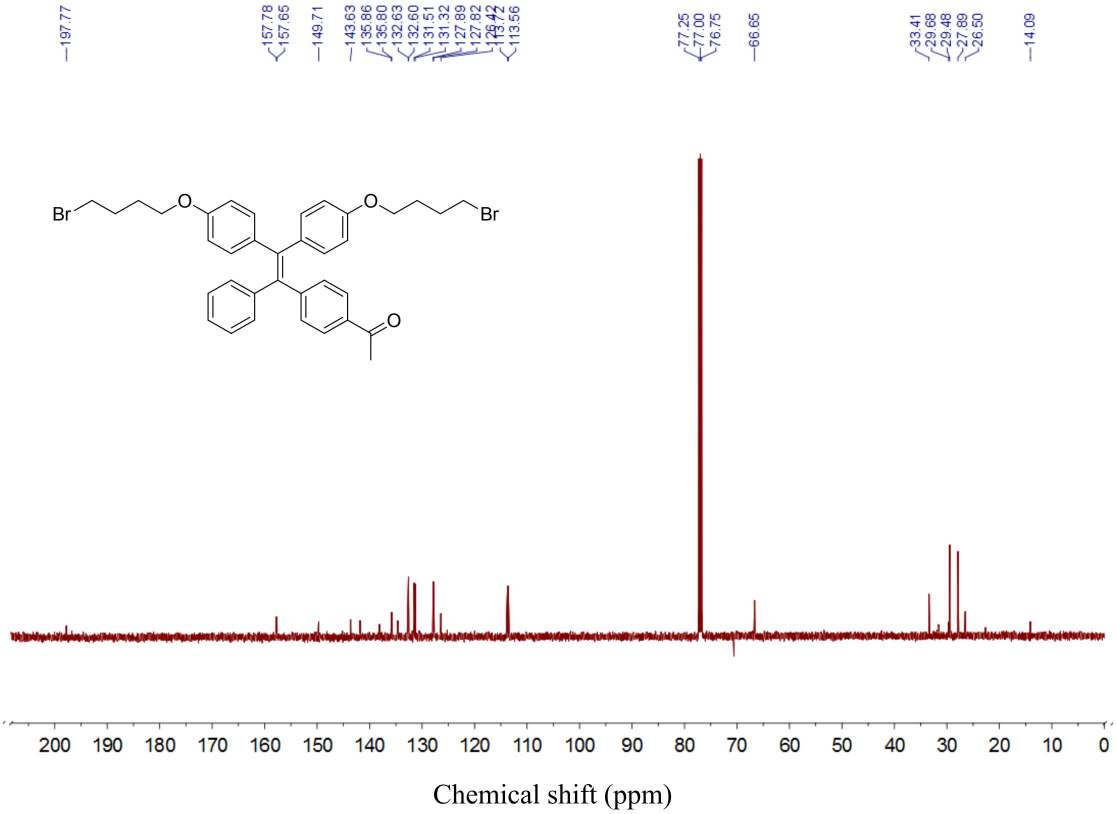


**Fig. S1** $^{1}H$ (top) and $^{13}C$ (bottom) NMR spectra of compound **4** in $CDCl_3$.

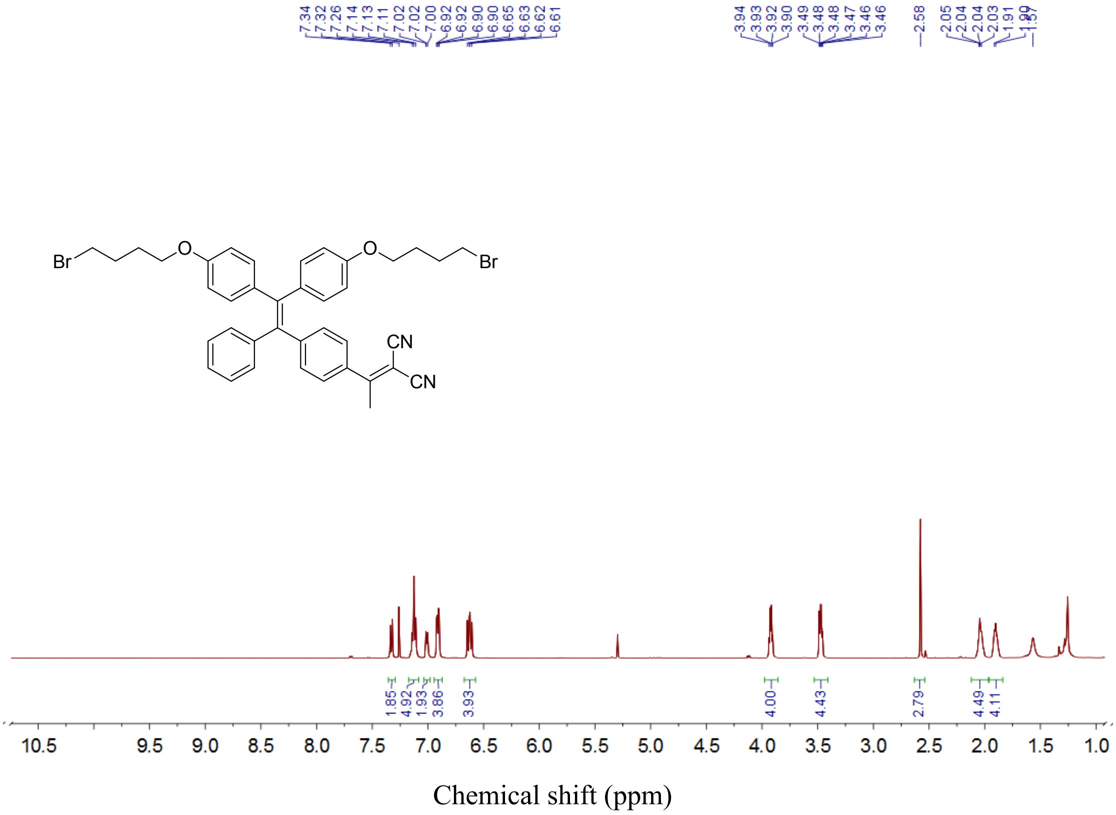

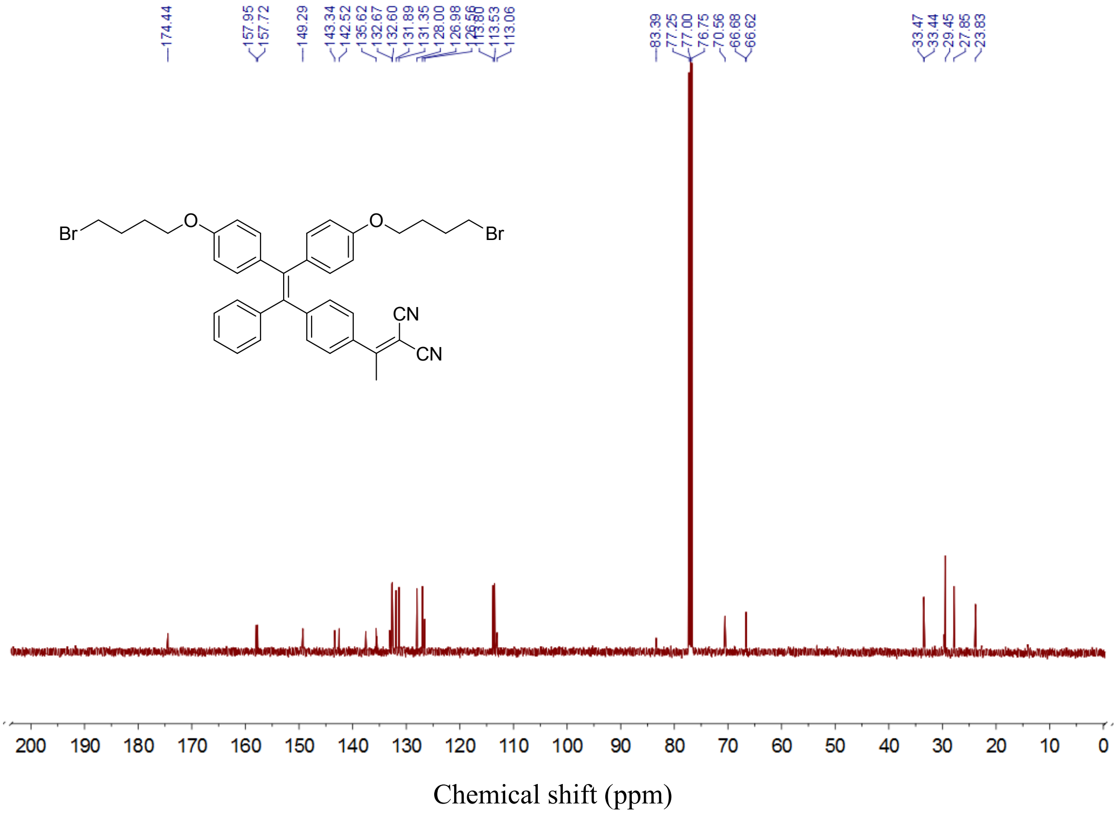


**Fig. S2** $^{1}$H (top) and $^{13}$C (bottom) NMR spectra of compound **TPECM-2Br** in $CDCl_3$.

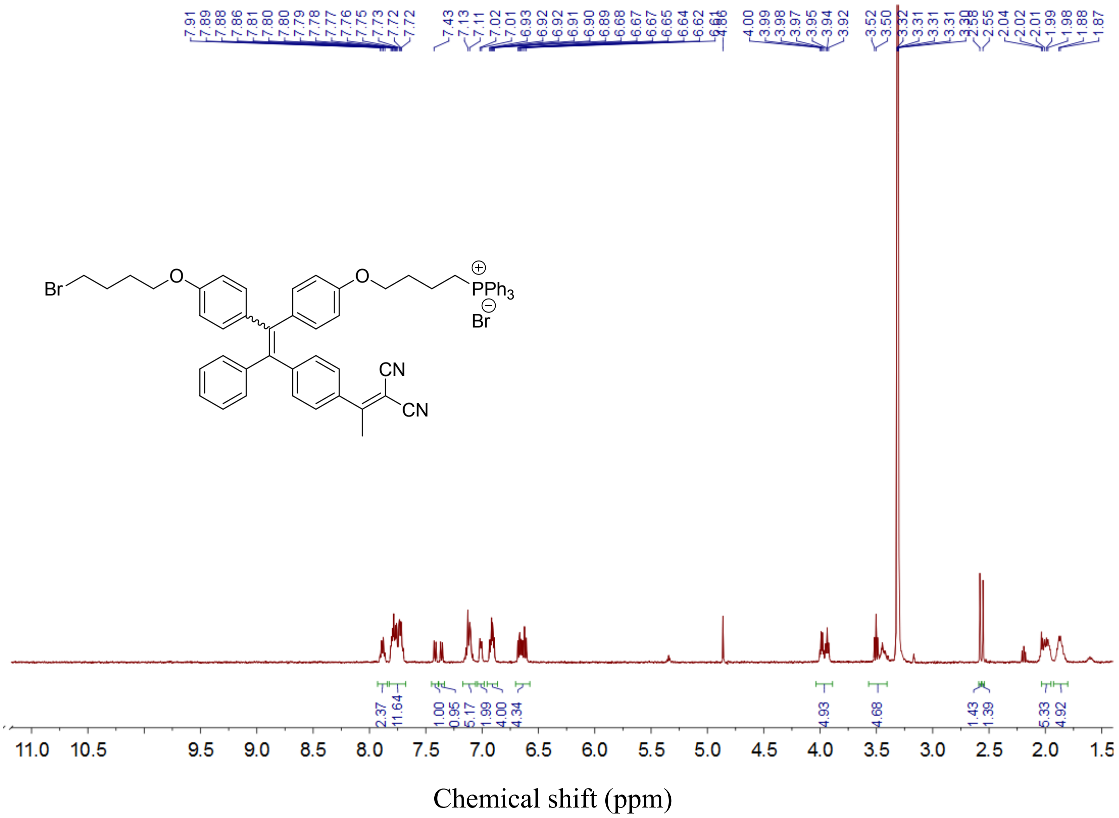

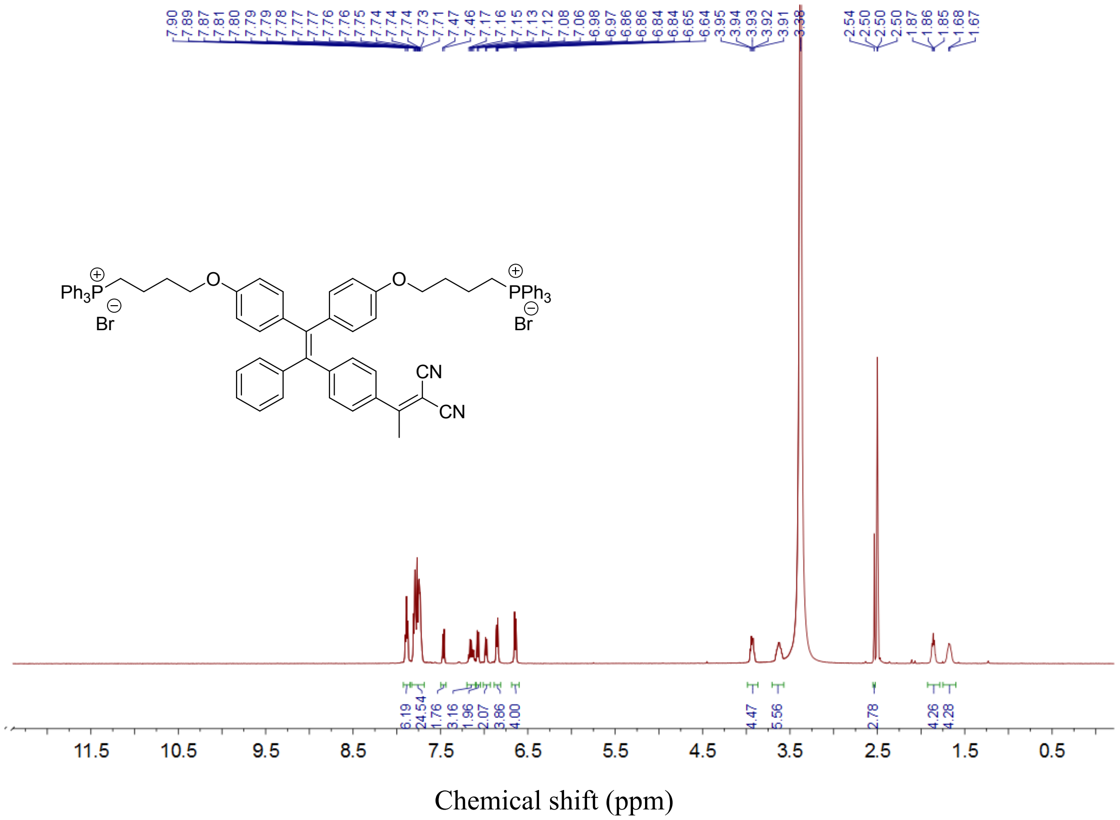

Fig. S3 $^{1}$H NMR spectra of compound **TPECM-1TPP** in Methanol-$d_4$ (top) and **TPECM-1TPP** (bottom) in DMSO-$d_6$.

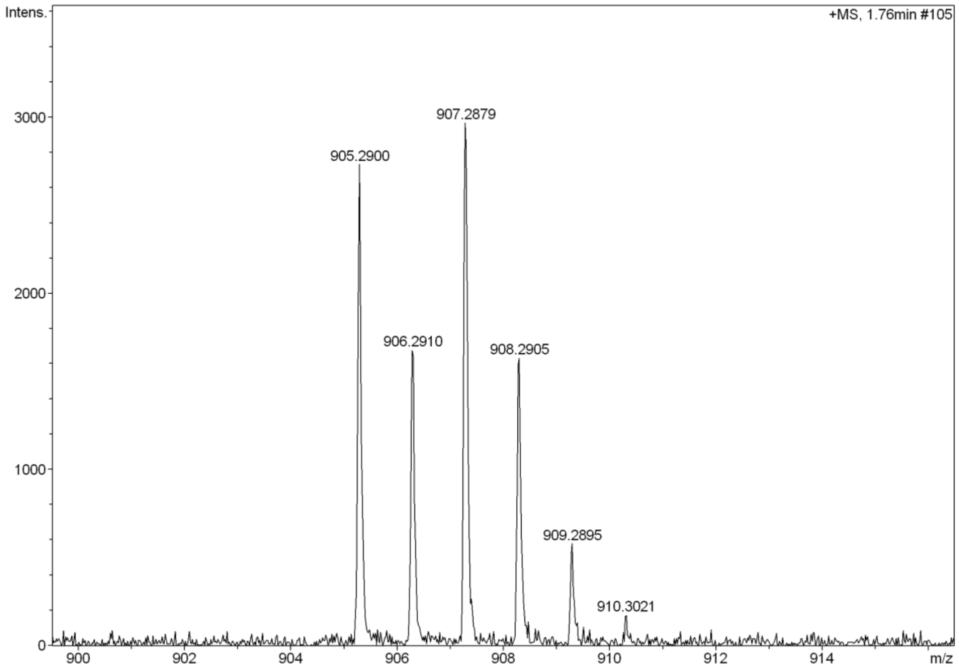

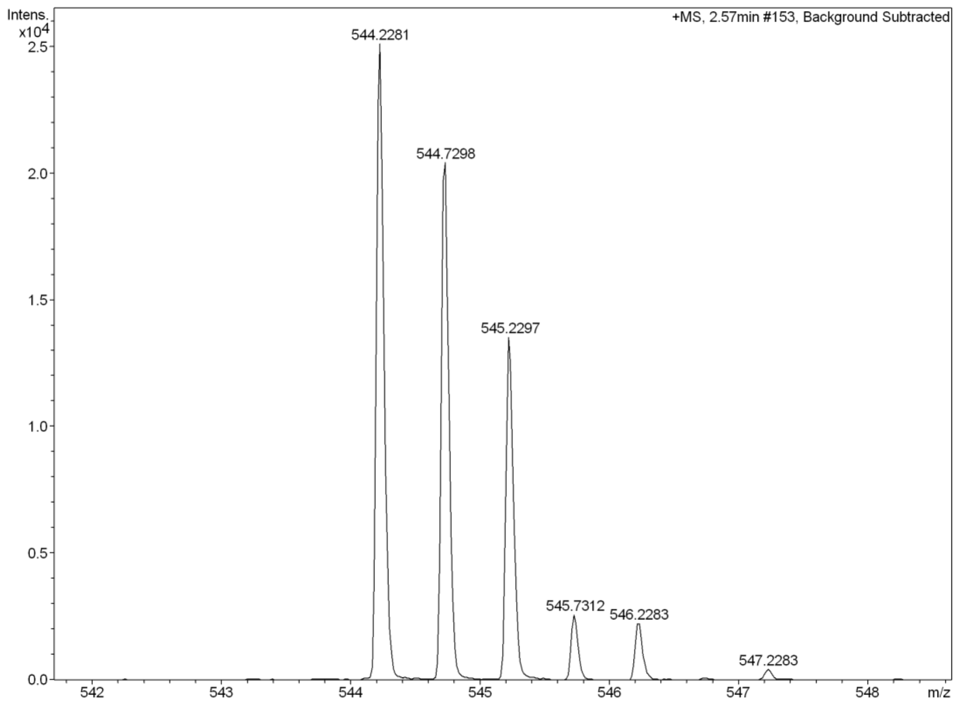


**Fig. S4** High Resolution mass spectroscopies (ESI) of **TPECM-1TPP** (top) and **TPECM-2TPP** (bottom).

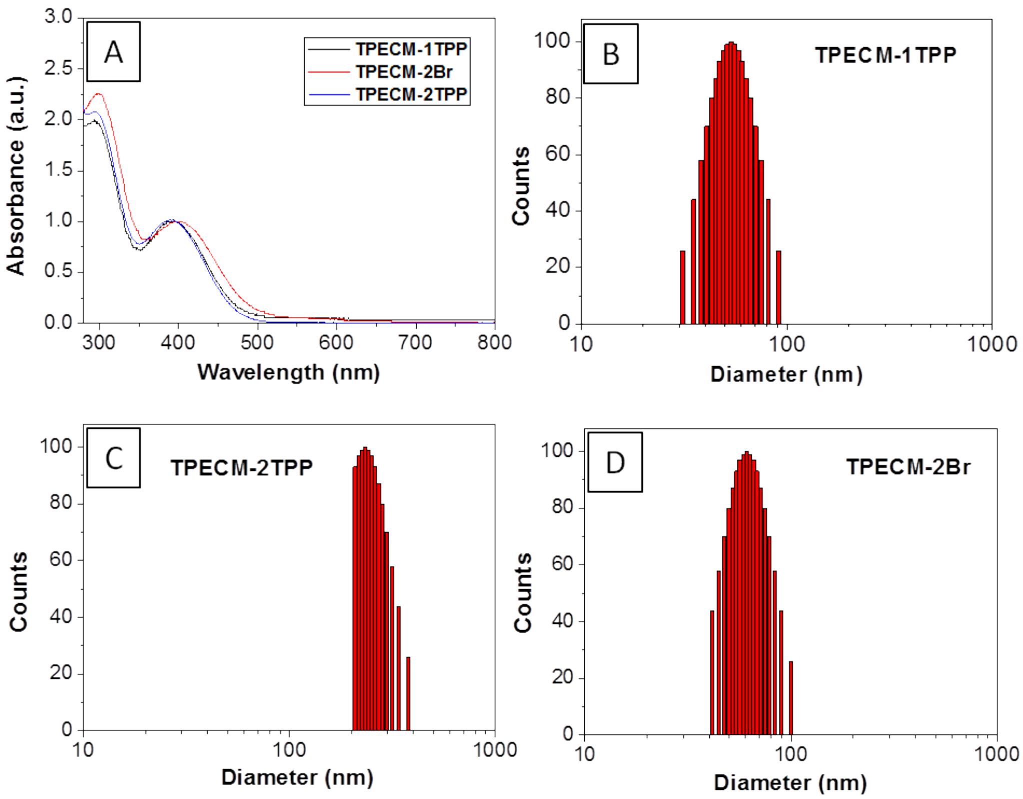


**Fig. S5** (A) Normalized UV-vis absorption spectra of **TPECM-2Br**, **TPECM-1TPP** and **TPECM-2TPP** in the mixture of DMSO/buffer (v/v = 1/199). Hydrodynamic diameters measured by laser light scattering (LLS) for (B) **TPECM-1TPP** (5 μM) in the mixture of DMSO/water (v/v = 1/99), (C) **TPECM-2TPP** (5 μM) in the mixture of isopropyl alcohol/hexane (v/v = 1/99), (D)

**TPECM-2Br** (5 μM) in the mixture of isopropyl alcohol/hexane (v/v = 1/99).

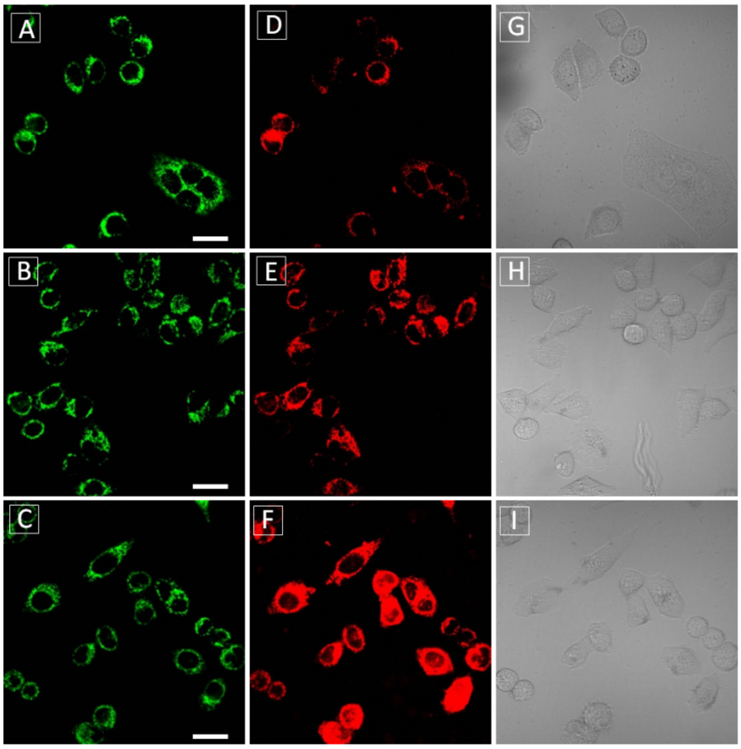


**Fig. S6** Confocal images of HeLa cells after treatment with **TPECM-1TPP** (1 μM) (top row, A, D, G), (2 μM) (middle row, B, E, H), and (5μM), (bottom row, C, F, I) for 3 hours, then the cells were co-stained with Mito-tracker green (200 nM). Images A, B and C are from Mito-tracker green, $\lambda_{ex}$ = 488 nm and $\lambda_{em}$ = 520 nm ± 20 nm; images D, E and F are from **TPECM-1TPP**, $\lambda_{ex}$ = 405 nm, $\lambda_{em}$ >560 nm long pass filter; images G, H and I are from bright field. All images share the same scale bar of 20 μm.

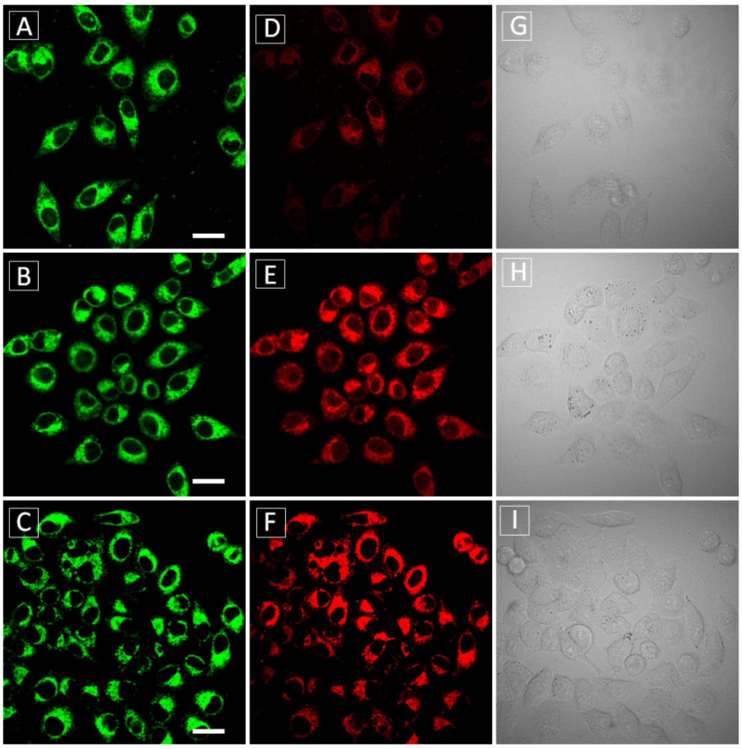


**Fig. S7** Confocal images of HeLa cells after treatment with **TPECM-2TPP** (1 μM) (top row, A, D, G), (2 μM) (middle row, B, E, H), and (5μM), (bottom row, C, F, I) for 3 hours, then the cells were co-stained with Mito-tracker green (200 nM). Images A, B and C are from Mito-tracker green, $\lambda_{ex}$ = 488 nm and $\lambda_{em}$ = 520 nm ± 20 nm; images D, E and F are from **TPECM-2TPP**, $\lambda_{ex}$ = 405 nm, $\lambda_{em}$ > 560 nm long pass filter; images G, H and I are from bright field. All images share the same scale bar of 20 μm.

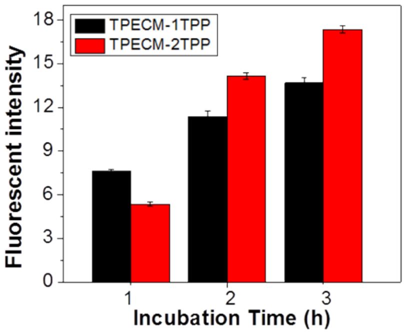


**Fig. S8** (A) Fluorescence intensity of HeLa cells upon incubation with **TPECM-1TPP** (5 μM) and **TPECM-2TPP** (5 μM) for different duration.

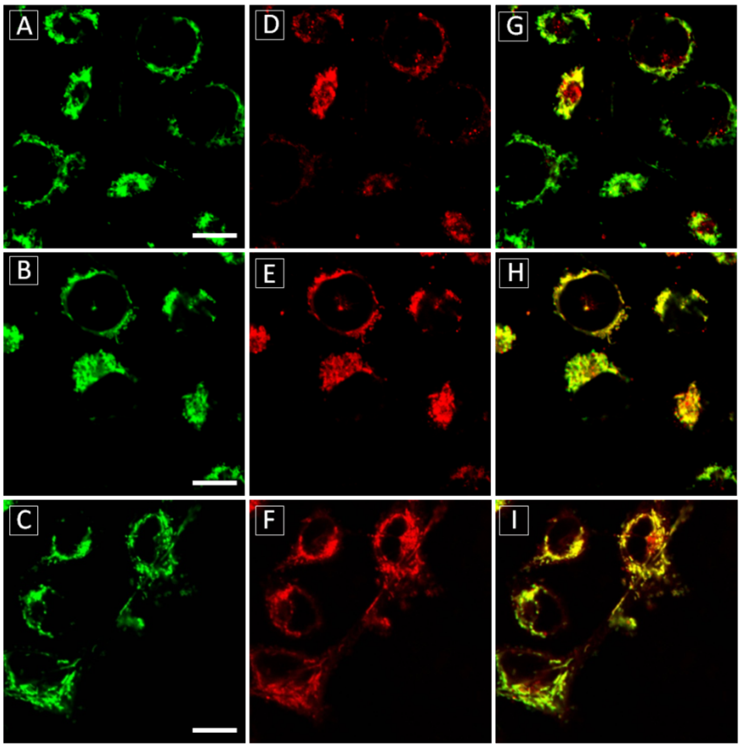


**Fig. S9** Confocal images of MDA-MB-231 cells after treatment with **TPECM-1TPP** (1 μM) (top row, A, D, G), (2 μM) (middle row, B, E, H), and (5μM), (bottom row, C, F, I) for 3 hours, then the cells were co-stained with Mito-tracker green (200 nM). Images A, B and C are from Mito-tracker green, $\lambda_{ex}$ = 488 nm and $\lambda_{em}$ = 520 nm ± 20 nm; images D, E and F are from **TPECM-1TPP**, $\lambda_{ex}$ = 405 nm, $\lambda_{em}$ >560 nm long pass filter; images G, H and I are from bright field. All images share the same scale bar of 20 μm.

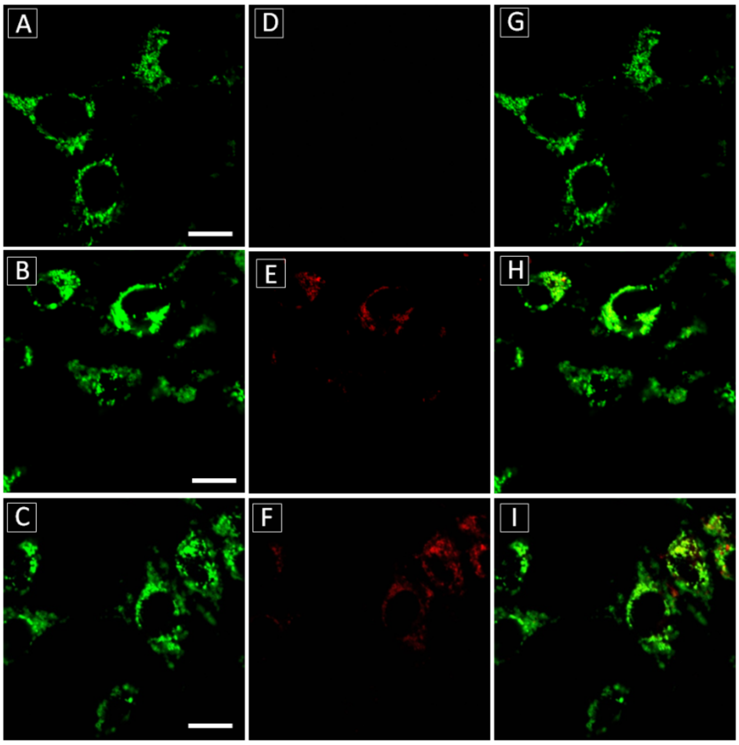


**Fig. S10** Confocal images of NIH-3T3 cells after treatment with **TPECM-1TPP** (1 μM) (top row, A, D, G), (2 μM) (middle row, B, E, H), and (5μM), (bottom row, C, F, I) for 3 hours, then the cells were co-stained with Mito-tracker green (200 nM). Images A, B and C are from Mito-tracker green, $\lambda_{ex}$ = 488 nm and $\lambda_{em}$ = 520 nm ± 20 nm; images D, E and F are from **TPECM-1TPP**, $\lambda_{ex}$ = 405 nm, $\lambda_{em}$ >560 nm long pass filter; images G, H and I are from bright field. All images share the same scale bar of 20 μm.

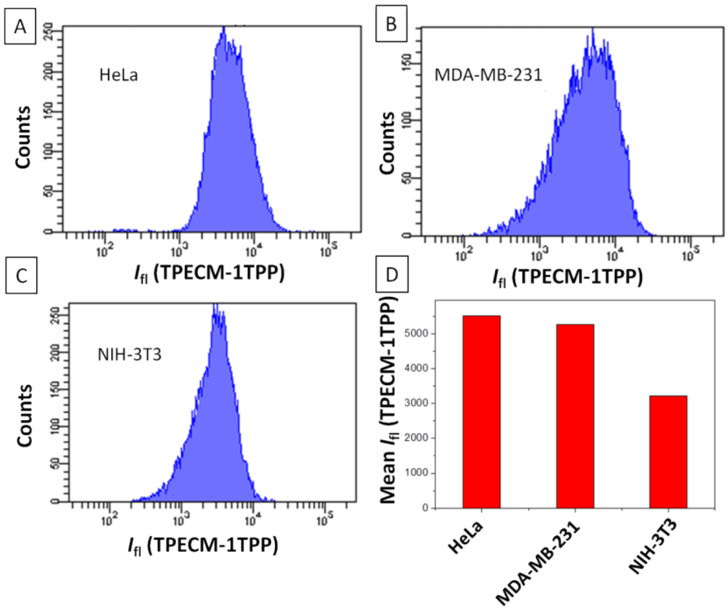


**Fig. S11** Flow cytometry of HeLa (A), MDA-MB-231 (B) and NIH-3T3 (C) cells after treatment with **TPECM-1TPP** (3 μM) for 2 hours; For each cell line, the total counted number of cells is 10,000. (D) Mean $I_{fl}$(TPECM-1TPP) for three different cell lines. $I_{fl}$(TPECM-1TPP) = fluorescence intensity of the probe **TPECM-1TPP**, $\lambda_{ex}$ = 405 nm; $\lambda_{ex}$ > 605 nm.

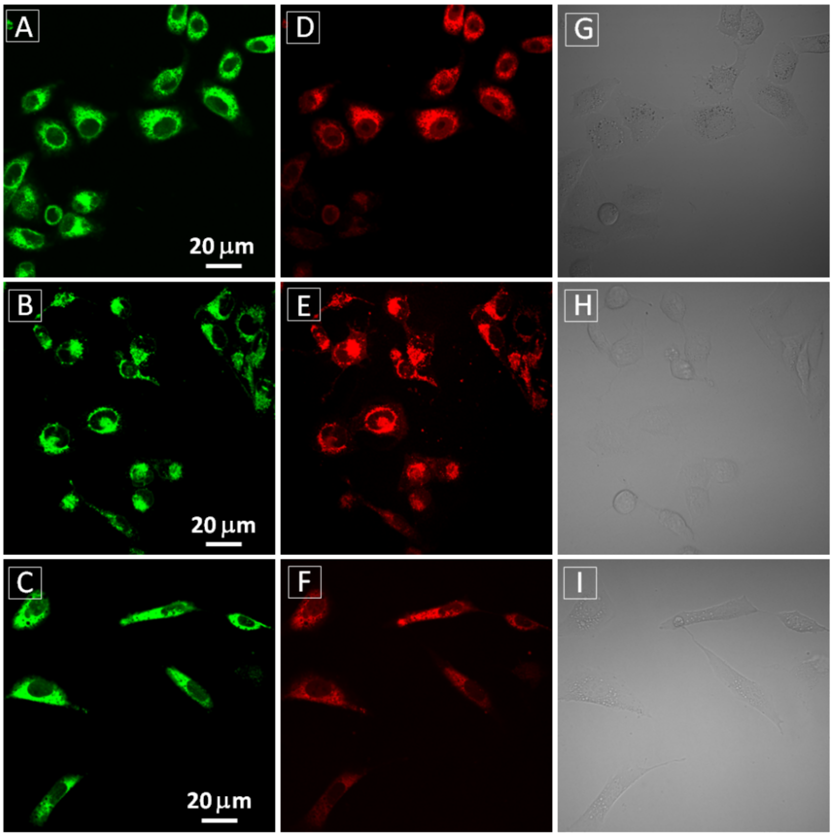


**Fig. S12** Fluorescence Images of HeLa cells (A, D, G), MDA-MB-231 (B, E, H) and NIH-3T3 (C, F, I) treated with 2 μM **TPECM-2TPP** for 3 hours, then the cells were co-stained with Mito-tracker green (200 nM). Images A, B and C are from Mito-tracker green, $\lambda_{ex}$ = 488 nm and $\lambda_{em}$ = 520 nm ± 20 nm; images D, E and F are from **TPECM-2TPP**, $\lambda_{ex}$ = 405 nm, $\lambda_{em}$ >560 nm long pass filter; images G, H and I are from bright field. All images share the same scale bar of 20 μm.

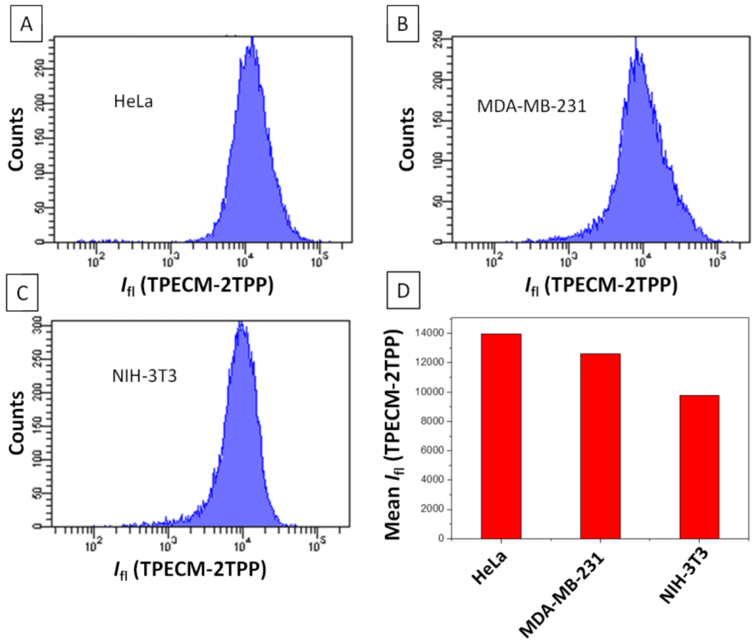

**Fig. S13** Flow cytometry of HeLa (A), MDA-MB-231 (B) and NIH-3T3 (C) cells after treatment with **TPECM-2TPP** (3 μM) for 2 hours; For each cell line, the total counted number of cells is 10,000. (D) Mean $I_{fl}$(TPECM-2TPP) for three different cell lines. $I_{fl}$(TPECM-2TPP) = fluorescence intensity of the probe **TPECM-2TPP**, $\lambda_{ex}$ = 405 nm; $\lambda_{ex}$ > 605 nm.

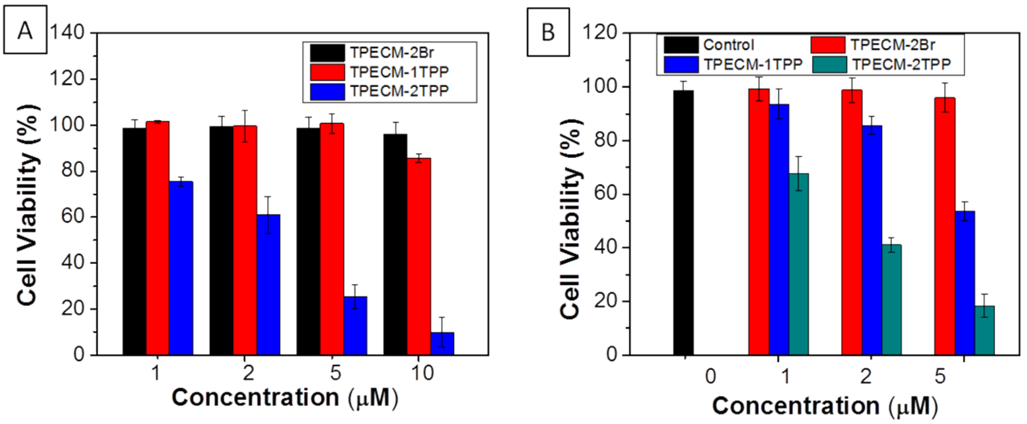


**Fig. S14** (A) The viability of MDA-MB-231 cells upon treatment with **TPECM-2Br**, **TPECM-1TPP** and **TPECM-2TPP** at different concentrations in dark for 24 h; (B) The viability of MDA-MB-231 cells upon treatment with **TPECM-2Br**, **TPECM-1TPP** and **TPECM-2TPP** at different concentrations for 3 h, which was followed by washing-away with PBS, light irradiation (0.10 W cm$^{-2}$, 8 min) and further incubation for 24 h.

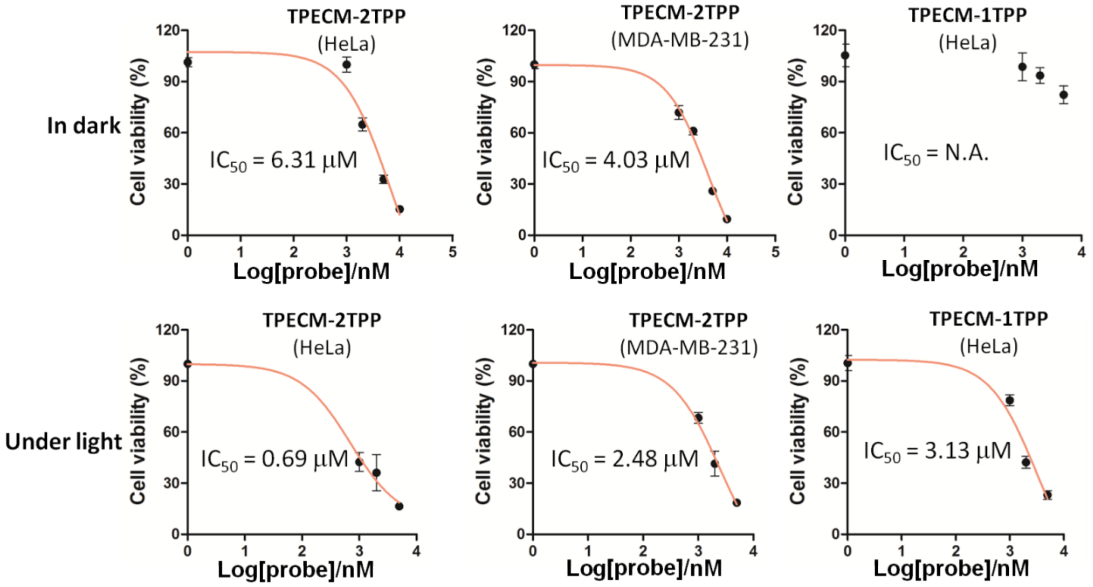


**Fig. S15** $IC_{50}$ of HeLa or MDA-MB-231 cells upon treatment with **TPECM-1TPP** or **TPECM-2TPP** in dark or under light irradiation, N.A. = not available.

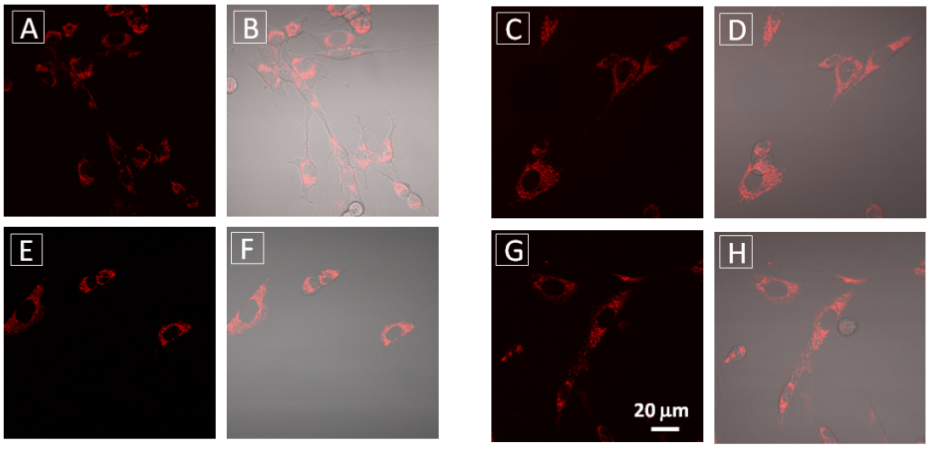


**Fig. S16** The mitochondria membrane potential of NIH-3T3 cells measured by tetramethyl rhodamine ethyl ester (TMRE) without treatment (A, B), with the treatment of 2 μM **TPECM-Br** (C, D), **TPECM-1TPP** (E, F) and **TPECM-2TPP** (G, H) for 3 h. A, C, E, G are images from the TMRE which was excited at 543 nm and detected at 575-625 nm. B, D, F and H are the overlay of TMRE image and bright field image. All images share the same scale bar of 20 μm.

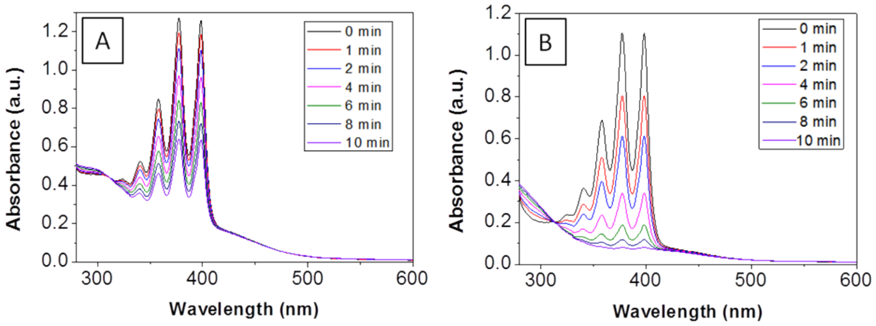


**Fig. S17** The Absorption spectra of ABDA (100 μM) in the presence of **TPECM-2Br** (20 μM) (A) and **TPECM-1TPP** (20 μM) (B) after different durations of white light irradiation.

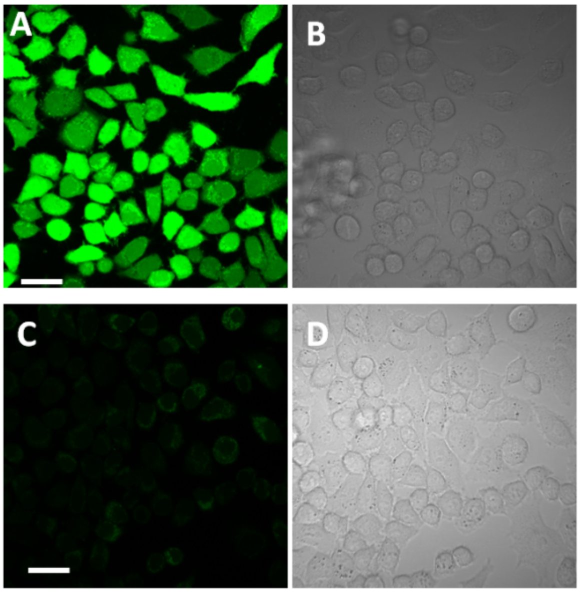


**Fig. S18** Detection of intracellular ROS production using DCF-DA after incubation of the cells with **TPECM-1TPP** (5 μM) for 3 h, followed by light irradiation (0.25 W $cm^{-2}$) (A and B) or in dark condition (C and D). Images A and C are from DCF-DA which was excited at 488 nm and detected at 510-560 nm. Images B and D are from the bright field. All images share the same scale of 20 μm.
